# IL-13 promotes functional recovery after myocardial infarction via direct signaling to macrophages

**DOI:** 10.1172/jci.insight.172702

**Published:** 2024-01-23

**Authors:** Santiago Alvarez-Argote, Samantha J. Paddock, Michael A. Flinn, Caelan W. Moreno, Makenna C. Knas, Victor A. Almeida, Sydney L. Buday, Amirala Bakhshian Nik, Michaela Patterson, Yi-Guang Chen, Chien-Wei Lin, Caitlin C. O’Meara

**Affiliations:** 1 Department of Physiology,; 2 Cardiovascular Research Center,; 3 Department of Cell Biology, Neurobiology, and Anatomy,; 4 Department of Pediatrics,; 5 Department of Microbiology and Immunology, and; 6 Department of Biostatistics, Medical College of Wisconsin, Milwaukee, Wisconsin, USA.

**Keywords:** Cardiology, Cytokines, Heart failure, Macrophages

## Abstract

There is great interest in identifying signaling pathways that promote cardiac repair after myocardial infarction (MI). Prior studies suggest a beneficial role for IL-13 signaling in neonatal heart regeneration; however, the cell types mediating cardiac regeneration and the extent of IL-13 signaling in the adult heart after injury are unknown. We identified an abundant source of IL-13 and the related cytokine, IL-4, in neonatal cardiac type 2 innate lymphoid cells, but this phenomenon declined precipitously in adult hearts. Moreover, IL-13 receptor deletion in macrophages impaired cardiac function and resulted in larger scars early after neonatal MI. By using a combination of recombinant IL-13 administration and cell-specific IL-13 receptor genetic deletion models, we found that IL-13 signaling specifically to macrophages mediated cardiac functional recovery after MI in adult mice. Single transcriptomics revealed a subpopulation of cardiac macrophages in response to IL-13 administration. These IL-13–induced macrophages were highly efferocytotic and were identified by high IL-1R2 expression. Collectively, we elucidated a strongly proreparative role for IL-13 signaling directly to macrophages following cardiac injury. While this pathway is active in proregenerative neonatal stages, reactivation of macrophage IL-13 signaling is required to promote cardiac functional recovery in adults.

## Abstract 2

<p>Myocardial infarction models and genetic approaches demonstrate that IL-13 stimulates distinct macrophages phenotypes and improves cardiac repair.</p>

## Graphical abstract

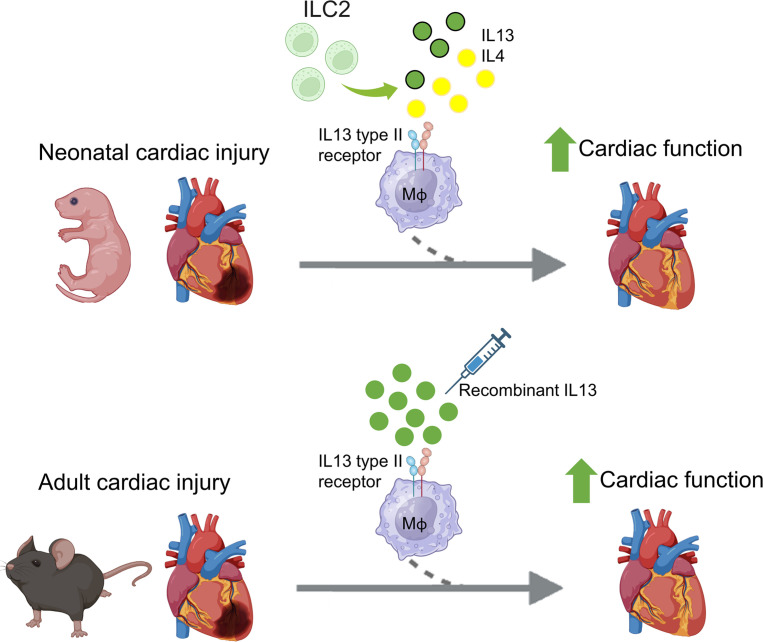

## Introduction

A body of literature supports a key role for macrophages in myocardial healing following injury. Their role extends to scenarios such as acute ischemia with and without reperfusion (1–4), cardiac transplantation (5–7), chronic heart failure (HF) (8, 9), and drug-induced cardiotoxicity (10), among others. In general, macrophages are critical components of the cardiac repair process, and their absence can result in healing defects, intramural thrombus formation, arrhythmias, and fatality (2, 3, 11–13).

Cardiac macrophages are a heterogeneous group of cells that can be embryonically or monocyte derived and are highly adaptable to the needs of their microenvironment (1, 3, 5, 6, 8, 14–17). Macrophage functions including angiogenesis, phagocytosis of dead cellular material, myofibroblast activation, and release of hypertrophic factors are generally thought to play a beneficial role in the heart after myocardial infarction (MI) (6, 11–13, 18–22). Conversely, cardiac macrophages can drive monocyte chemotaxis and excessive fibrosis, resulting in adverse outcomes after injury (5, 6, 19, 23). While broad macrophage classes in the heart have been recently well characterized, we lack a comprehensive understanding of the specific signaling pathways that regulate cardiac macrophage functions — namely, few signaling stimuli that promote a reparative cardiac macrophage phenotype — have been extensively described.

Our group routinely employs the neonatal mouse model of heart regeneration to identify proreparative cell types and mechanisms that can be reactivated in adults to promote cardiac repair after MI. We recently demonstrated that global deletion of IL-13 or global deletion of 1 subunit of the IL-13 receptor, IL-4 receptor α (IL-4Rα), inhibit cardiac regeneration in neonatal mice (24, 25). This neonatal heart regeneration deficit was only partially recapitulated by IL-4Rα deletion in cardiomyocytes (CMs) (25), indicating that other cell types are involved. IL-13 is a cytokine that is classically attributed to type II immunity across tissues (26, 27). It has been well studied in the context of hyperreactive airway disease, where it is known to act on various pulmonary cell types — including smooth muscle, epithelial, and alveolar macrophages — to promote mucus production, eosinophilia, bronchial constriction, and interstitial fibrosis (26). IL-13 also promotes eosinophil chemotaxis as well as IgE and mucus production in the gut in response to helminthic infections (26), and it promotes interstitial fibrosis across multiple organs by acting directly on tissue-resident fibroblasts (26, 28). More recently, an emerging yet small body of literature has linked IL-13 to the cardiac injury response in adults. Observational studies in humans showed that IL-13 serum levels correlate with improved ventricular function in ischemic patients with HF (29), while levels of the type II IL-13 receptor heterodimer, IL-4Rα/IL-13Rα1, are decreased in patients with HF (30). In adult mice, IL-13 deletion modestly aggravated ischemic HF (31). Considering the global gene deletion strategies employed in prior animal studies, the cardiac cell types mediating the IL-13 response and the extent by which IL-13 directly influences cardiac repair after MI in the adult heart have not been defined.

Here, we elucidate a strongly proreparative role for macrophage IL-4Rα signaling in neonatal mice — a model capable of endogenous cardiac regeneration. Expression of 2 cytokines that activate IL-4Rα, IL-13 (via the type II [IL-4Rα/IL-13Rα1] receptor) and IL-4 (via the type I [IL-4Rα/γc] or type II receptor), could be readily detected by type 2 innate lymphoid cells (ILC2s) in the neonatal heart. However, cellular production of these cytokines as well as frequency of cardiac ILC2s declined precipitously into adulthood. Reactivation of the type II receptor in adults by recombinant IL-13 (rIL-13) administration significantly improved cardiac function after MI. While multiple cardiac cell types in the adult heart can respond to IL-13, improved cardiac function was mediated exclusively by rIL-13 signaling directly to macrophages, illustrating a remarkable role for exogenous IL-13 signaling in the post-MI injury response. Our work defines the utility of employing the neonatal model of heart regeneration to identify proreparative mechanisms that can be leveraged to improve outcomes after MI in adults.

## Results

### Macrophage deletion of IL-4Rα impairs cardiac functional recovery following neonatal MI.

We previously demonstrated that global deletion of either the IL-4/13 receptor subunit, IL-4Rα (IL-4Rα^–/–^), or global deletion of IL-13 both impaired cardiac regeneration in neonatal mice (24, 25). Deletion of IL-4Rα specifically in CMs only partially recapitulated impaired regeneration phenotypes, such as decreased CM cell cycle activity (25), observed in IL-4Rα^–/–^ mice, suggesting that IL-4Rα expression on other cell types mediates the neonatal cardiac injury response. We measured expression of IL-4Rα on multiple cardiac cell types in the neonatal heart after MI and found the highest expression of IL-4Rα in cardiac macrophages (Supplemental Figure 1, A–D; supplemental material available online with this article; https://doi.org/10.1172/jci.insight.172702DS1). Therefore, we hypothesized that IL-4Rα deletion on macrophages improves neonatal cardiac repair.

**Figure 1:**
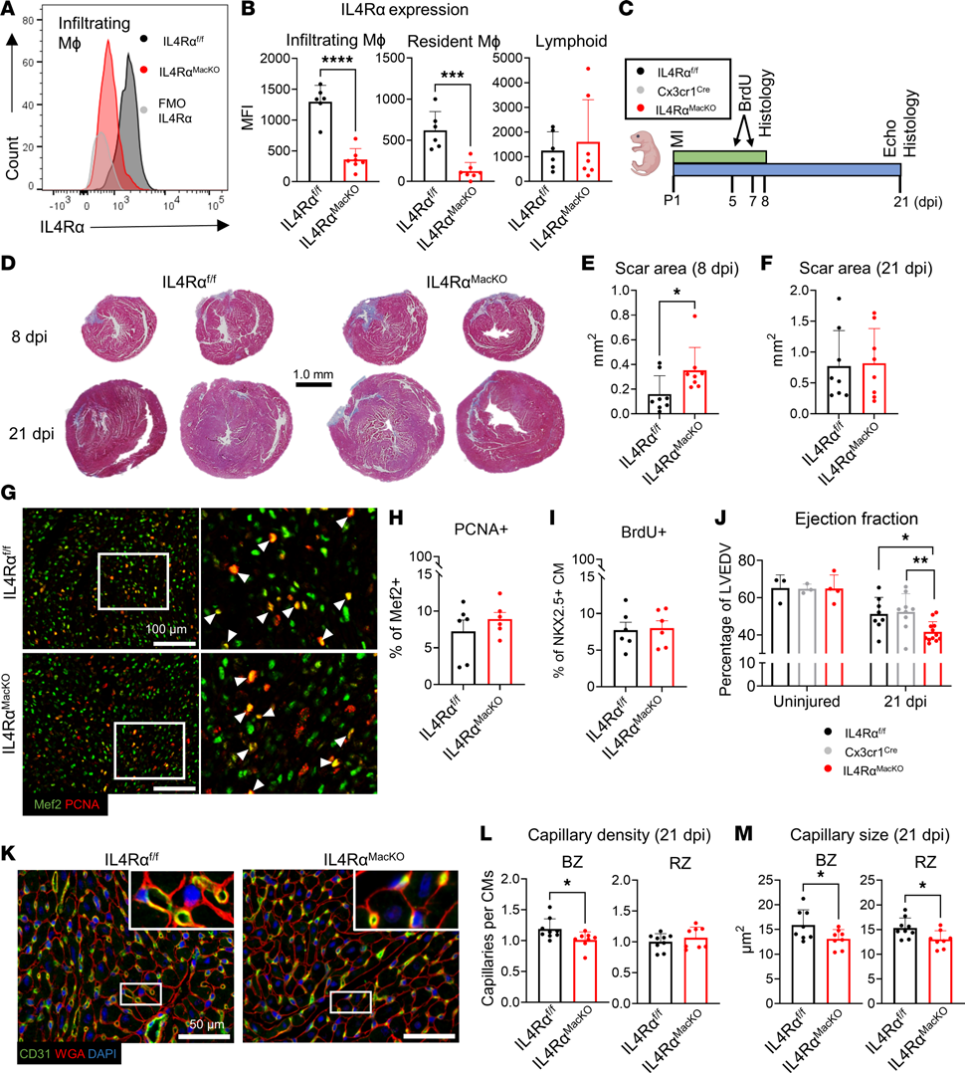
Macrophage (Mφ) IL-4Rα deletion impairs neonatal cardiac repair. (**A**) Representative histogram plots of IL-4Rα expression in cardiac Mφ isolated from IL-4Rα^fl/fl^ and IL-4Rα^MacKO^ mice at 4 dpi after P1 MI. FMO–IL-4Rα control is also shown. (**B**) Quantification of IL-4Rα MFI in infiltrating Mφ, resident Mφ, and lymphoid cells. (**C**) Experimental scheme of P1 MI, BrdU injections on 5 and 7 dpi, heart collections for histology at 8 dpi, cardiac echocardiogram (Echo), and heart collection for histology at 21 dpi. (**D**) Representative images showing Gömöri trichrome staining of mouse hearts at 8 and 21 dpi. Scale bar: 1 mm. (**E** and **F**) Quantification of total scar area in mm^2^ of mice at 8 dpi and 21 dpi. (**G**) Representative images of Mef2/PCNA immunostaining of cardiac sections at 8 dpi after P1 MI. Arrowheads indicate PCNA^+^/Mef2^+^ cells. White box inset indicates zoomed-in region. (**H**) Quantification of PCNA^+^/Mef2^+^ cells as a percentage of total Mef2^+^ nuclei quantified. (**I**) Quantification of BrdU^+^/NKX2.5^+^ cells (staining not shown) as a percentage of total Nkx2.5^+^ nuclei quantified. (**J**) Quantification of LV ejection fraction of mice at 21 dpi after P1 MI or age-matched uninjured conditions. (**K**) Representative images of CD31, WGA, and DAPI staining of mouse hearts at 21 dpi after P1 MI. (**L** and **M**) Quantification of capillary density and capillary size at the BZ and RZ of mouse hearts at 21 dpi. Data are presented as mean ± SD. Each data point represents 1 mouse. *P < 0.05, **P < 0.01, ***P < 0.001, ****P < 0.0001. Comparison by unpaired 2-tailed *t* test in **B**, **E**, **F**, **H**, **I**, **L**, and **M**. Interaction of condition (uninjured versus 21 dpi) and genotype effect by 2-way ANOVA and Sidak’s post hoc test for IL-4Rα^MacKO^ versus other 2 genotypes in **J**.

We crossed IL-4Rα^fl/fl^ with Cx3cr1^Cre^ mice to generate IL-4Rα^fl/fl^;Cx3cr1^Cre^ (herein referred to as IL-4Rα^MacKO^) whereby IL-4Rα is depleted specifically in macrophages (32). We confirmed IL-4Rα deletion in both infiltrating (CCR2^+^Timd4^–^) and tissue-resident (CCR2^–^Timd4^+^) cardiac macrophages at 4 days post injury (dpi) in P1 pups (Figure 1, A and B, and Supplemental Figure 1, E and F). CCR2 and Timd4 were used to define infiltrating versus resident macrophages as a combination of these markers have been previously employed to characterize these populations in lineage-tracing experiments (3, 5, 6, 16, 19). As expected, lymphoid cells showed no detectable change in IL-4Rα expression (Figure 1B and Supplemental Figure 1F).

To test the role of macrophage IL-4Rα in neonatal heart regeneration, we subjected P1 mice to MI followed by histological analysis (8 or 21 dpi) and echocardiography (21 dpi) (Figure 1C). IL-4Rα^MacKO^ mice had significantly larger scars toward the cardiac apex compared with controls at 8 dpi (Figure 1, D and E), suggesting either increased sensitivity to MI or exaggerated scarring. Interestingly, we found no difference in scarring between genotypes at 21 dpi (Figure 1, D and F) and no difference in CM DNA synthesis or cell cycle activity (Figure 1, G–I), indicating that IL-4Rα deletion in macrophages does not prevent CM DNA synthesis, cell cycle activity, or scar size over the course of the regenerative period but perhaps exacerbates scarring at early time points after injury. While there was no difference in scarring at 21 dpi, nor CM proliferation between genotypes, IL-4Rα^MacKO^ mice had significantly lower percent ejection fraction (%EF) compared with controls (IL-4Rα^fl/fl^ or Cx3cr1^Cre^) (Figure 1J), indicating that, despite normal CM proliferation, lack of IL-13 signaling to cardiac macrophages has a deleterious effect on cardiac function after neonatal MI. Importantly, there was no difference in cardiac function between uninjured animals, indicating that impaired %EF in IL-4Rα^MacKO^ is in response to cardiac injury.

Macrophages that promote cardiac regeneration are thought to be highly angiogenic (11, 18, 19). IL-4Rα^MacKO^ hearts had reduced capillary density at the border zone (BZ) but not remote zone (RZ) (Figure 1, K and L) and a smaller capillary cross-sectional area in both regions (Figure 1, K and M). Taken together, these results indicate that, while IL-4Rα deletion in macrophages does not influence CM regeneration, it exacerbates scar area early after MI, impairs capillary density, and impairs cardiac function after injury in neonates.

### IL-4 and IL-13 expression in the heart declines from neonatal to adult stages.

**Figure 2:**
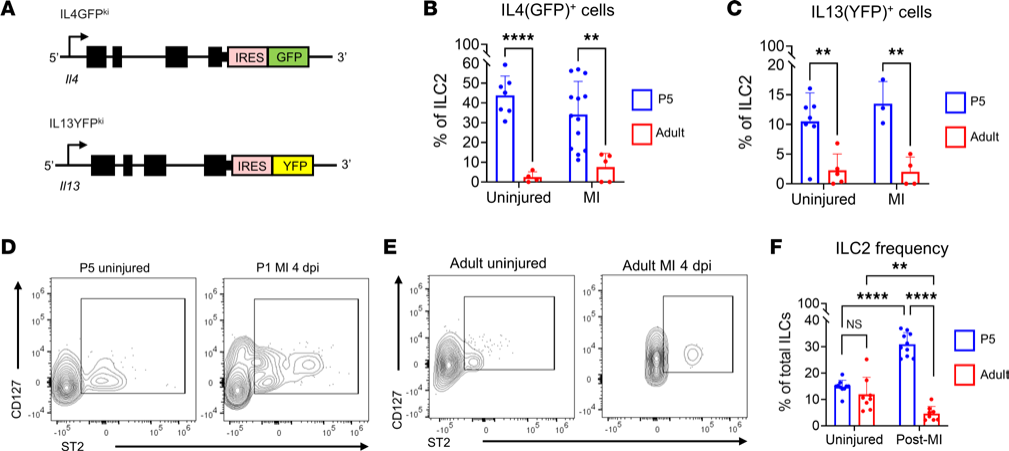
IL-4 and IL-13 expression is enriched in neonatal ILC2s. (**A**) Schematic of IL-4GFP^KI^ and IL-13YFP^KI^ transgenic mouse line alleles. (**B**) Quantification of GFP^+^ cells in P5 versus adult mice in uninjured and at 4 dpi after MI. GFP^+^ cells in IL-4GFP^KI/WT^ mice were normalized to WT age-matched littermates. (**C**) Quantifications of YFP^+^ cells in P5 versus adult mice in uninjured and at 4 dpi. YFP^+^ cells in IL-13YFP^KI/WT^ mice were normalized to WT age-matched littermates. (**D** and **E**) Representative contour plots of CD127 versus IL-1RL1 in cardiac non–B/T lymphoid cells in neonatal (**D**) and adult (**E**) mouse hearts. (**F**) Quantification of ILC2 frequency in P5 versus adult mouse hearts either uninjured or at 4 dpi. Data are shown as mean ± SD. Each data point represents 1 mouse. **P < 0.01, ****P < 0.0001. Age effect by 2-way ANOVA and Sidak’s post hoc test for uninjured and after MI conditions in **B** and **C**. Interaction effect of age and condition by 2-way ANOVA and Sidak’s post hoc test in **F**.

Expression of signaling pathway components that promote neonatal heart repair have been shown to decrease into adulthood, correlating with a decline in cardiac repair potential (33). We tested if production of the 2 cytokines known to activate IL-4Rα, IL-13 and IL-4, also decline from neonatal to adult stages. Initially, we attempted to measure IL-13 expression in whole heart homogenates by immunoblotting; however, given the very low expression levels of IL-13 and IL-4 in the whole heart (34), we could not detect any clear band in heart samples from neonatal or adult mice (data not shown). To more sensitively test IL-13 and IL-4 production by cardiac cell types, we used reporter mouse lines whereby GFP or YFP are knocked in (KI) to the 3′ untranslated region of the IL-4 (IL-4GFP^KI/WT^) and IL-13 (IL-13YFP^KI/WT^) endogenous loci; IL-4– and IL-13–producing cells express GFP and YFP, respectively (Figure 2A). We measured expression of GFP and YFP in cell types previously reported to produce IL-4/13, including myeloid cells, T cells, B cells, and LC2 using the gating strategy shown in Supplemental Figure 2A (35–37). In neonatal mice, we detected expression of GFP and YFP predominantly in ILC2s (CD45^+^CD11b^–^CD90.2^+^CD127^+^ST2^+^) in both uninjured hearts and in hearts at 4 dpi following P1 MI (Figure 2, B and C, and Supplemental Figure 2, B–E), with ~40% of ILC2s GFP^+^ (IL-4^+^) and ~12% YFP^+^ (IL-13^+^). While we detected ILC2s in the adult heart, the frequency of this cell type (as a percentage of non–T cell/B cell lymphoid cells) declined significantly compared with frequency observed in neonatal hearts. Furthermore, ILC2s in the adult heart were minimally GFP^+^ or YFP^+^ (Figure 2, B and C), and we could not detect any robust source of either GFP or YFP (Supplemental Figure 2, B–E) in adults in either the uninjured setting or after MI (Figure 2, D–F). Thus, in the neonatal heart, ILC2s are highly abundant and are actively secreting IL-4Rα–related cytokines, and both ILC2 frequency and production of IL-4 and IL-13 decline from neonatal to adult stages.

### rIL-13 administration promotes cardiac functional recovery in adult mice after MI.

**Figure 3:**
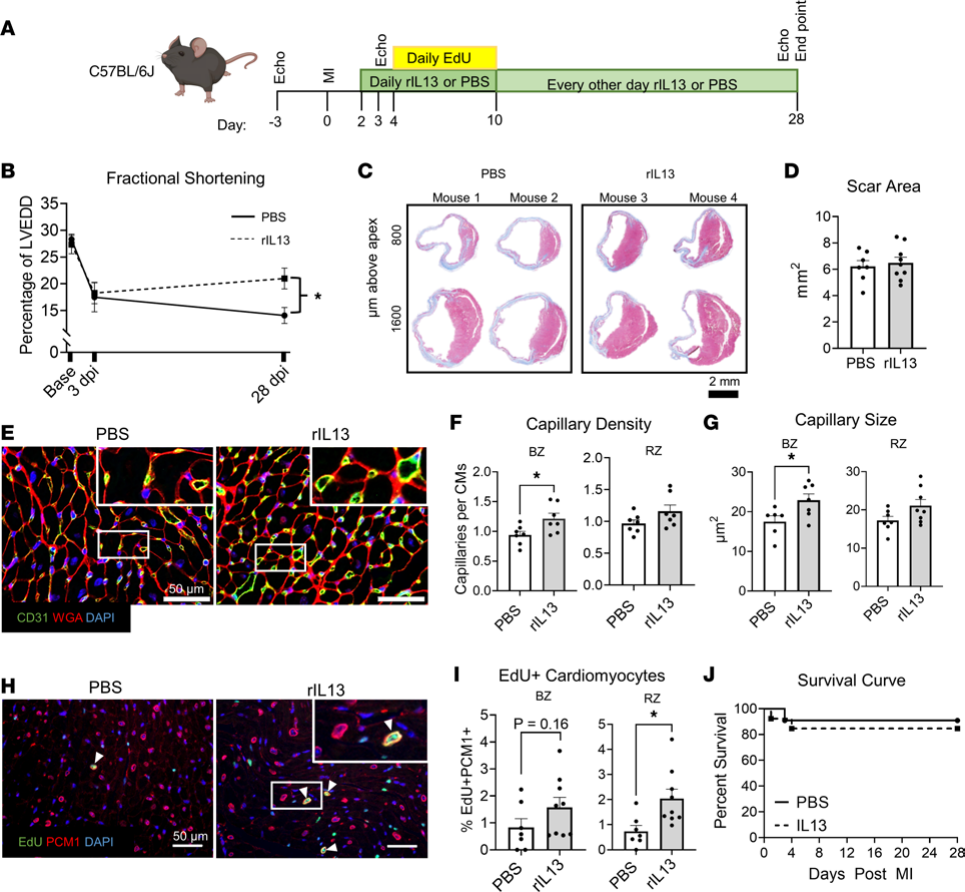
rIL-13 administration improves functional recovery after MI in adult mice. (**A**) Experiment protocol. (**B**) Fractional shortening shown as percentage of LV end-diastolic diameter (LVEDD). (**C**) Representative images of Gömöri trichrome staining of mouse heart sections at 28 dpi. Scale bar: 2 mm. (**D**) Quantification of total scar area summed from 3 serial cardiac sections. (**E**) Representative images of CD31, WGA, and DAPI of 28 dpi hearts. White box insets indicate zoomed-in regions. (**F** and **G**) Quantifications of capillary density and capillary size at the BZ and RZ in 28 dpi heart sections. (**H**) Representative images of EdU, PCM1, and DAPI in cardiac sections from 28 dpi mice. (**I**) Quantifications of EdU^+^PCM1^+^ cells as a percentage of total PCM1^+^ cells in BZ and RZ. (**J**) Survival curves up to 28 dpi after MI. Data are shown as mean ± SD. Each data point represents 1 mouse. *P < 0.05. Interaction of treatment (PBS versus rIL-13) and time effect by 2-way repeated-measures ANOVA and Sidak’s post hoc test at 28 dpi in **B**. Comparison by unpaired 2-tailed *t* test in **D**, **F**, **G**, and **I**. Data were analyzed by Mantel-Cox in **J**.

IL-4Rα can heterodimerize with the common γ chain (IL-2rg, γc) forming the type I receptor (IL-4Rα/γc) that can be activated by IL-4, or it can heterodimerize with IL-13Rα1 to form the type II receptor (IL-4Rα/IL-13Rα1) that can be activated by either IL-13 or IL-4 (38). Thus, while IL-4 can activate both receptors, IL-13 can only activate the type II receptor. Considering IL-13 deletion impairs neonatal cardiac repair (24, 25) and cardiac abundance of IL-4 and IL-13 that can both activate the type II receptor decrease in the adult heart, we next tested if reactivation of the type II receptor (IL-4Rα/IL-13Rα1) could improve cardiac functional recovery in adults after injury. To target the type II receptor, we administered murine recombinant IL-13 (rIL-13) or vehicle (PBS) to adult C57BL/6J mice following MI (Figure 3A). EdU was administered to track cell DNA synthesis. At 3 dpi, animals receiving either rIL-13 or PBS showed a comparable initial decline in fractional shortening (%FS) at the midpapillary level indicating similar injury size. While cardiac function of mice receiving PBS showed no improvement from 3 to 28 dpi, mice receiving rIL-13 showed a trend toward partial functional recovery. Thus, %FS of rIL-13–treated mice was significantly higher than PBS-injected mice at 28 dpi (Figure 3B). Although FS improved with rIL-13 administration, we found no difference in scar size at 28 dpi (Figure 3, C and D), indicating that rIL-13 improves function of the surviving myocardial tissue but does not resolve scarring. Consistent with neonatal experiments, rIL-13 administration increased capillary density and capillary cross-sectional area in the BZ (Figure 3, E–G) but not statistically in the RZ.

IL-13 stimulates neonatal CM cell cycle activity (24, 34), and consistent with these studies, rIL-13 administration increased CM EdU incorporation after MI in adults; this was significant in the RZ (Figure 3, H and I). While cardiac function was improved, rIL-13 administration did not significantly influence survival after MI (Figure 3J). Taken together, IL-4Rα/IL-13Rα1 activation by systemic rIL-13 administration improved cardiac functional recovery after MI in adult mice, correlating with increased capillary density and size, and increased CM DNA synthesis. While these experiments do not distinguish the cell types mediating improved cardiac function, based on our neonatal experiments, we hypothesized that macrophages (Figure 1), or potentially CMs (25), mediate this effect.

### rIL-13 signaling to CMs does not mediate functional recovery after MI.

**Figure 4:**
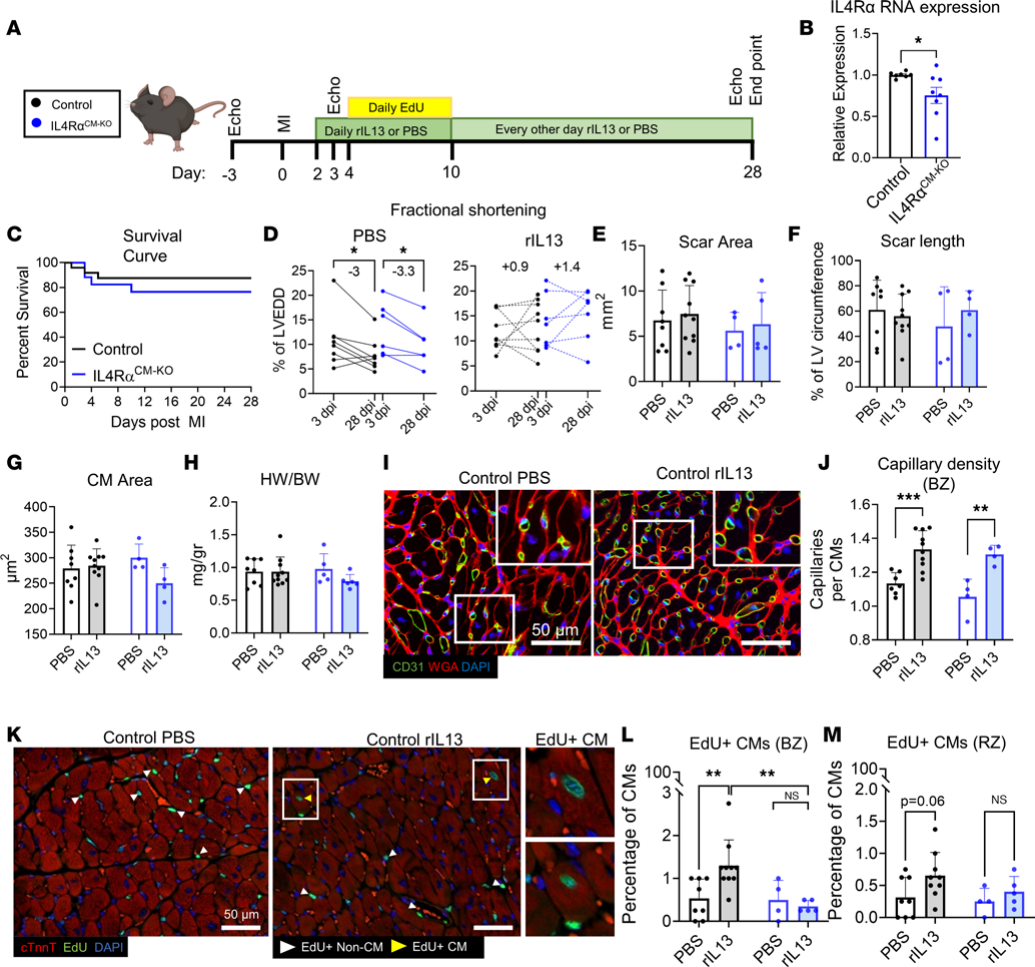
rIL-13 signaling to CMs promotes CM cell cycle activity but does not mediate functional recovery after MI in adult mice. (**A**) Experiment protocol. Group colors in the inset are used throughout entire figure. (**B**) Quantification of IL-4Rα mRNA expression from adult mouse whole LV tissue. (**C**) Survival curve up to 28 dpi after MI. (**D**) Quantification of fractional shortening (%FS) at 3 and 28 dpi. (**E** and **F**) Quantification of scar area as absolute mm^2^ and scar length in 28 dpi hearts. (**G**) Quantification of CM cross-sectional area at the BZ. (**H**) Quantification of HW/BW ratios at 28 dpi. (**I**) Representative images of CD31, WGA, and DAPI staining of 28 dpi cardiac tissue sections. White box inset indicates zoomed-in region. Scale bar: 50 μm. (**J**) Capillary density at the BZ from 28 dpi cardiac tissue sections. (**K**) Representative images of EdU, cardiac troponin T (cTnnt), and DAPI staining of cardiac sections at 28 dpi. Scale bar: 50 μm. (**L** and **M**) Quantification of EdU^+^ CMs (cTnnt^+^) at the BZ and RZ. Data are shown as mean ± SD. Each data point represents 1 mouse. *P < 0.05, **P < 0.01, ***P < 0.001. Data compared by 2-tailed, unpaired *t* test in **B**, by Mantel-Cox in **C**, and by 2-way ANOVA and Sidak’s post hoc comparison in **D**. Interaction effect between treatment and genotype by 2-way ANOVA and Sidak’s post hoc comparison between PBS versus rIL-13 treatment for control mice and between Control versus IL-4Rα^CM-KO^ mice treated with rIL-13 in **L**.

IL-4rα deletion in CMs (IL-4Rα^fl/fl^;Myh6^Cre^, referred to as IL-4Rα^CM-KO^) decreases CM cell cycle activity and partially impairs cardiac regeneration in neonates (25), and rIL-13 administration to adult mice increases CM DNA synthesis. We next tested if rIL-13 signaling directly to CMs mediates functional recovery after MI (Figure 4A). While IL-4Rα is expressed at relatively low levels in adult CMs, we detected decreased RNA expression in hearts of IL-4Rα^CM-KO^ mice (Figure 4B), confirming gene deletion in our model. There was no difference in post-MI survival between control (IL-4Rα^fl/fl^) and IL-4Rα^CM-KO^ mice (Figure 4C). While mice from either genotype treated with PBS showed a progressive decline in %FS from 3 to 28 dpi (Figure 4D), mice from of both genotypes responded similarly to rIL-13 administration, demonstrating a trend toward improved %FS from 3 to 28 dpi (Figure 4D). We observed no difference in scar size (Figure 4, E and F), CM cross-sectional area (Figure 4G), or heart weight/body weight (HW/BW) ratio between genotypes or treatment groups (Figure 4H). rIL-13 administration increased capillary cross-sectional area and density, regardless of genotype (Figure 4, I and J). Interestingly, while rIL-13 increased CM DNA synthesis in control animals, this response was completely abrogated in IL-4Rα^CM-KO^ mice (Figure 4, K–M), demonstrating that rIL-13 can signal directly to adult CMs to promote DNA synthesis, but unlike in neonates, CMs do not mediate cardiac repair nor improve function in response to rIL-13 after MI. A limitation to this experiment is that we did not include Myh6^Cre^ controls, and we are acutely aware that Myh6^Cre^ alone can substantially influence CM phenotypes and cardiac physiology (39). While it is possible that diminished EdU incorporation is a result of Myh6^Cre^, this is unlikely considering that elevated EdU is a direct consequence of rIL-13 administration.

### rIL-13 signaling to macrophages mediates functional recovery after MI.

**Figure 5:**
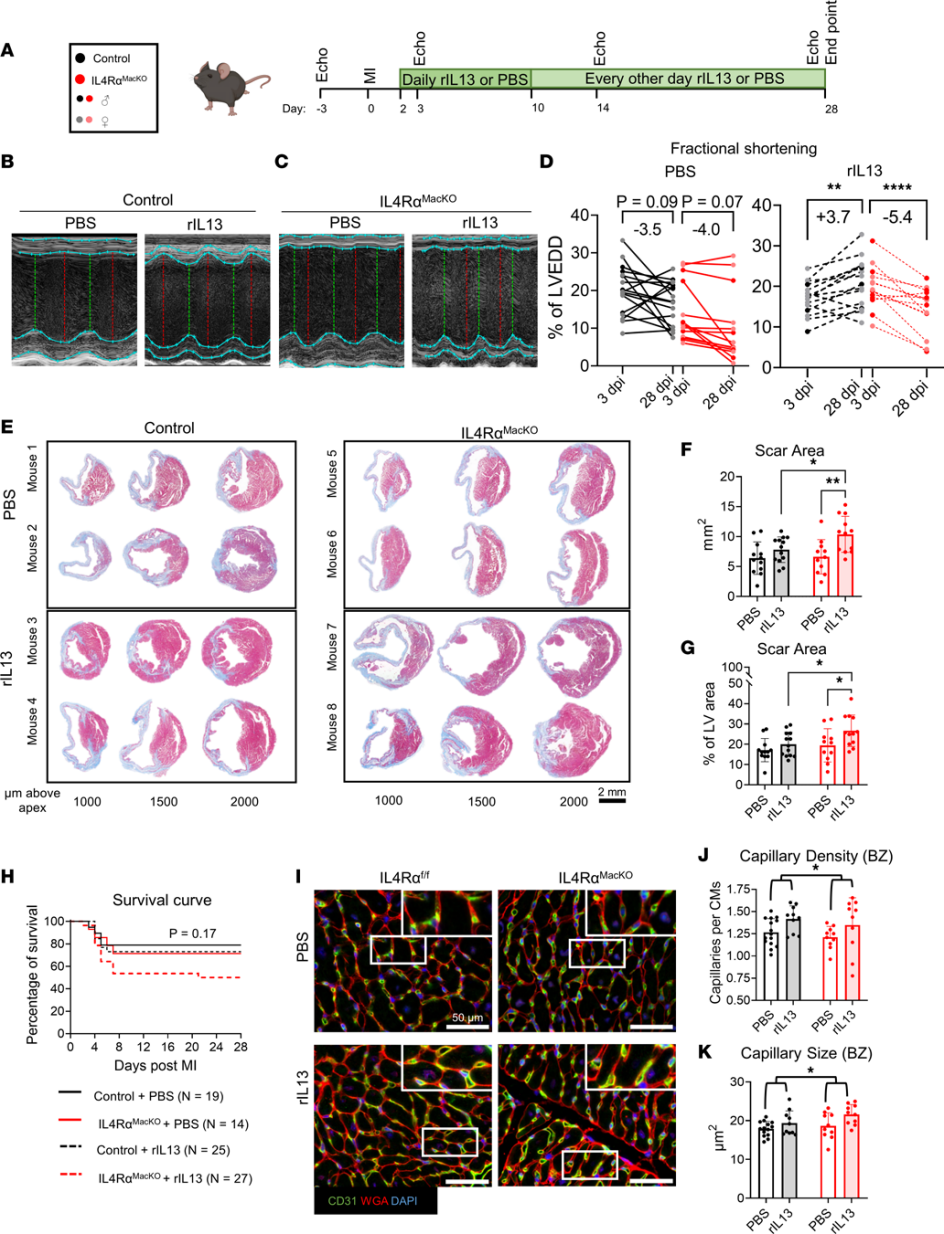
rIL-13 signaling to macrophages mediates functional recovery after MI in adult mice. (**A**) Experiment protocol. Group colors in the inset are used throughout entire figure. (**B** and **C**) Representative echocardiogram short-axis M-mode images at midpapillary level from control and IL-4Rα^MacKO^ mouse hearts. (**D**) Quantification of %FS at 3 and 28 dpi of control and IL-4Rα^MacKO^ mice after MI and PBS versus rIL-13 administration. (**E**) Representative images of Gömöri trichrome staining of mouse heart sections at 28 dpi. Scale bar: 2 mm. (**F** and **G**) Quantification of total scar area as absolute mm^2^ and percentage of total LV area. (**H**) Kaplan-Meier survival curves. (**I**) Representative images of CD31, WGA, and DAPI staining of 28 dpi cardiac tissue sections. White box inset indicates zoomed-in regions. Scale bar: 50 μm. (**J** and **K**) Quantifications of capillary density and capillary size at the BZ in 28 dpi heart sections. Data are shown as mean ± SD. Each data point represents 1 mouse. Darker data points represent male mice in **D**. *P < 0.05, **P < 0.01, **** *P* < 0.0001. Time effect by 2-way RM ANOVA for control and IL-4Rα^MacKO^ treated with PBS in **D**. Interaction effect of time and genotype by 2-way RM ANOVA and Sidak’s post hoc comparison in **D**. Treatment effect by 2-way ANOVA and Sidak’s post hoc comparison in **F** and **G**. Comparison by Mantel-Cox in **H**. Treatment effect by 2-way ANOVA in **I** and **J**.

We next asked if rIL-13 signaling to macrophages mediates the functional recovery observed after MI in adult mice. We subjected adult IL-4Rα^MacKO^ and control mice to MI, followed by PBS or rIL-13 administration (Figure 5A). Both IL-4Rα^fl/fl^ and Cx3cr1^Cre^ control mice responded similarly to rIL-13 after MI (Supplemental Figure 3); therefore, we pooled data from these genotypes for our littermate control group. While body weights were not different between groups (Supplemental Figure 4A), at 28 dpi, rIL-13 administration resulted in significantly larger hearts and HW/BW ratios only in IL-4Rα^MacKO^ mice compared with control (Supplemental Figure 4, B and C). In agreement with HW/BW findings, cardiac volume measured by echocardiography showed that IL-4Rα^MacKO^ had more dilated hearts at 28 dpi than control mice only after MI (Supplemental Figure 4, D and E), suggesting than lack of IL-13/IL-4Rα macrophage activation after MI has an aggravating effect on cardiac remodeling and dilation. Interestingly, IL-4Rα^MacKO^ mice treated with PBS showed a trending decrease in %FS at 3 dpi, suggesting increased sensitivity to MI in IL-4Rα^MacKO^ mice. On the other hand, following MI, control and IL-4Rα^MacKO^ mice treated with rIL-13 showed a similar decline in %FS at 3 dpi (Supplemental Figure 4F). Regardless of genotype, mice treated with PBS showed no improvement in %FS from 3 to 28 dpi, since both groups showed a –3.5% to –4% change in %FS (Figure 5, B and D). On the other hand, control mice treated with rIL-13 showed an approximately 3.7% recovery in %FS from 3 to 28 dpi (Figure 5, B and D, and Supplemental Figure 4F), comparable with that observed in C57BL/6J mice treated with rIL-13 (Figure 3B). However, IL-4Rα^MacKO^ mice treated with rIL-13 showed no recovery in %FS from 3 to 28 dpi; they showed a change of –5.4% in %FS (Figure 5, C and D, and Supplemental Figure 4F). We analyzed the change in %FS normalized to the initial decline (i.e., ΔFS = [%FS at 28 dpi – %FS at 3 dpi]/[%FS at baseline – %FS at 3 dpi]) and confirmed that control mice treated with rIL-13 had a recovery of approximately 40% of the initial decline, while PBS-treated mice and IL-4Rα^MacKO^ mice treated with rIL-13 showed a progressive decline of 50% (Supplemental Figure 4G). These functional data indicate that improved %FS in response to rIL-13 administration is mediated by direct signaling to macrophages. Interestingly, we found no improvement in %EF after IL-13 administration to either control or IL-4Rα^MacKO^ mice; nonetheless, rIL-13 administration ameliorated this decline in control mice but not in IL-4Rα^MacKO^ mice (Supplemental Figure 4H). Of note, %EF is measured on the longitudinal axis of the heart, which summarizes cardiac contractility, including contractile myocardium and noncontractile scar. %FS is based on a cross-sectional measurements at the mid-papillary level of the heart proximal to the ischemic region and, therefore, measures cardiac contractility at a level where myocardium is not replaced by noncontractile scar. These results suggest that IL-13/IL-4Rα macrophage activation improves cardiac contractility after MI by influencing contractility of the viable myocardium.

While rIL-13 administration did not influence scar size in control mice, surprisingly, IL-4Rα^MacKO^ mice treated with rIL-13 had significantly larger scars (nearly 50% larger) compared with control rIL-13–treated mice and compared with IL-4Rα^MacKO^ mice treated with PBS (Figure 5, E–G). Increased total scar area is driven by increases in both scar length and thickness (Supplemental Figure 4, I and J), whereas total myocardium area or septal wall thickness was not different between any groups (Supplemental Figure 4, K and L). These data indicate that rIL-13 signals to other cardiac cell types besides macrophages to aggravate scarring, a phenotype only exhibited when IL-4Rα is deleted from macrophages. Interestingly, while there was no significant difference in survival after MI between any of the genotypes or treatment groups, we found a trend toward decreased survival following MI in IL-4Rα^MacKO^ mice treated with rIL-13 (*P* = 0.17) (Figure 5H), again suggesting that rIL-13 signals to a nonmacrophage cell types to aggravate post-MI outcomes (which is only apparent when IL-4Rα is deleted from macrophages), clearly illustrating a strongly proreparative role for rIL-13 signaling directly to macrophages. Mice dying in the weeks after MI include those that had suffered the most severe injuries (Supplemental Figure 4M); thus, we speculate that the larger scar size and reduced %FS in IL-4Rα^MacKO^ mice treated with rIL-13 would only be exacerbated, had all animals survived to the end point. In agreement with our observations in WT mice, rIL-13 administration increased capillary density and size in control mice at the BZ (Figure 5, I–K). This increase was also seen in IL-4Rα^MacKO^ mice, indicating that capillary phenotypes in rIL-13–treated adult animals are not mediated by macrophages and do not correlate with functional outcomes or aggravated scar phenotypes.

Collectively, these results elucidate a complex role for rIL-13 administration after MI. Global rIL-13 administration is overall beneficial to cardiac function after MI, but rIL-13 administration in the absence of macrophage IL-4Rα/IL-13Rα1 is detrimental, resulting in larger scars after MI and a trend toward decreased survival compared with vehicle treatment alone. Thus, the presence of the type II receptor on macrophages is vital not only for rIL-13–mediated improvements in cardiac function but also for protection against excessive scar formation.

### Characterization of a macrophage subpopulation activated by rIL-13.

**Figure 6:**
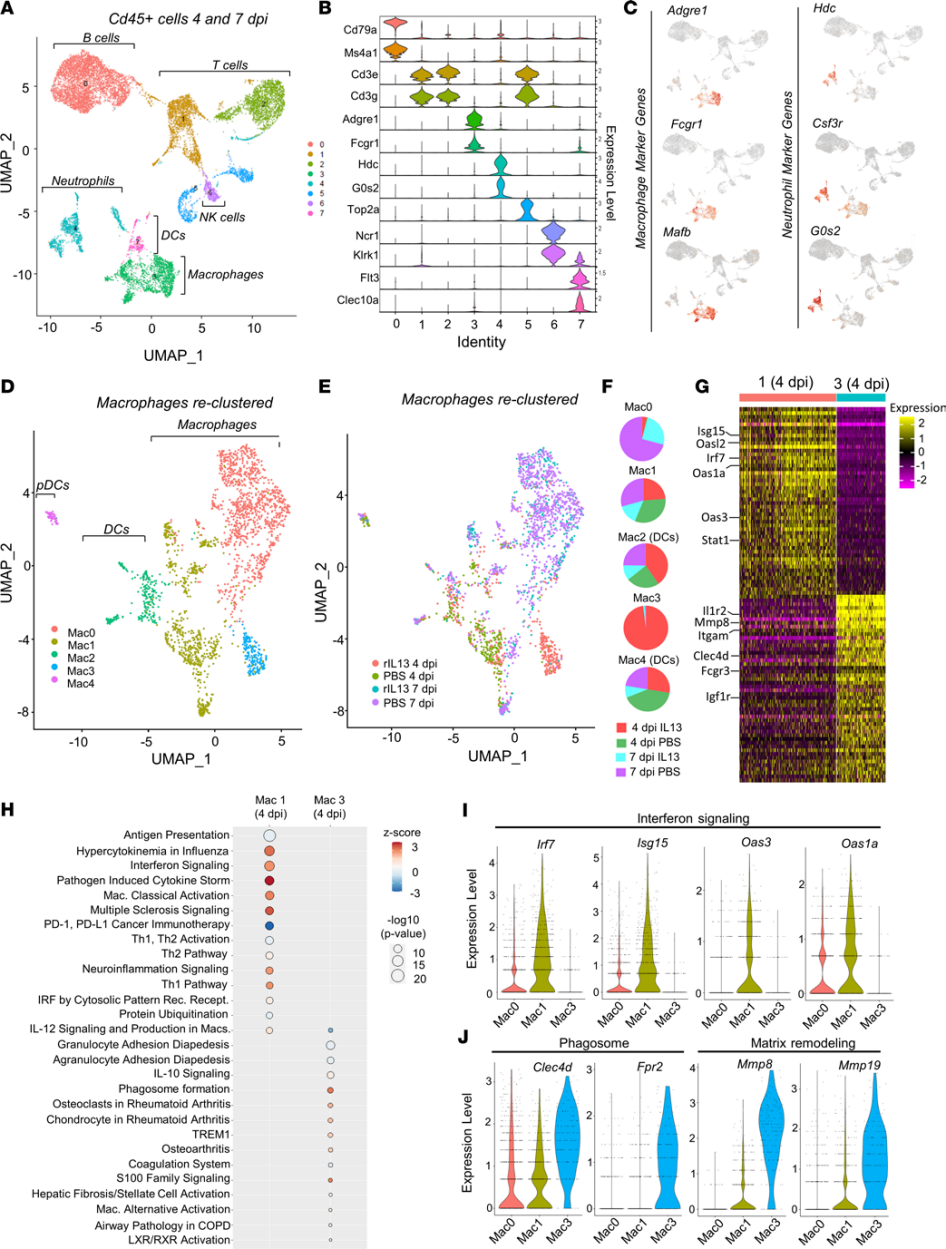
rIL-13 induces a unique macrophage cluster at 4 dpi. (**A**) Uniform Manifold Approximation and Projection (UMAP) plot of live leukocytes isolated from mouse hearts at 4 and 7 dpi after PBS or rIL-13 daily administration. Four samples were given: PBS at 4 dpi, PBS at 7 dpi, rIL-13 at 4 dpi, rIL-13 at 7 dpi. *n* = 2 or 3 mice per sample. (**B**) Violin plots showing expression of unique markers that defined the identity of each of the 7 clusters identified in **A**. (**C**) Feature plots showing expression of macrophage specific markers in clusters 3 and 7 and unique neutrophils markers in cluster 4. (**D**) Clusters 3 and 7 from **A** were reclustered, resulting in 5 unique clusters (clusters 0–4), among which 0, 1, and 3 correspond to macrophages and 2 and 4 are DCs. (**E** and **F**) UMAP plots and pie charts showing the contribution of each sample to each macrophage cluster. Mac3 is 98% derived from rIL-13 4 dpi. (**G**) Heatmap showing top 50 DEG in Mac1 and Mac3 at 4 dpi only. Top colors represent cluster ID. Color scale bar represents directionality of expression (log FC threshold 0.7. Adjusted *P* < 0.1 × 10^–17^). (**H**) Ingenuity pathway analysis identified top upregulated canonical pathways in macrophage clusters 1 and 3. Color scale bar represents directionality of expression. Circle size represents statistical significance. (**I**) Violin plots showing expression of IFN response elements in macrophage clusters 0, 1, and 3. (**J**) Violin plots showing expression of phagocytic associated markers and extracellular matrix remodeling factors in macrophage clusters 0, 1, and 3.

We next characterized macrophage phenotypes in the heart following rIL-13 administration to gain further insights into the mechanisms underlying its effects on cardiac repair. We performed MI on adult C57BL/6J mice and administered rIL-13 or PBS by daily i.p. injection starting at 2 dpi, and isolated cardiac leukocytes (CD45^+^) by FACS at 4 and 7 dpi for single-cell RNA-Seq (scRNA-Seq). In total, we sequenced 13,126 leukocytes (3,062 from 4 dpi rIL-13; 2,136 from 4 dpi PBS; 3,598 from 7 dpi rIL-13; 4,330 from 7 dpi PBS) at an average read depth of ~90,000 reads per cell. Cluster analysis of all cells revealed 8 main populations consisting of B cells (cluster 0: *Cd79a*, *Ms5a1*), T cells (clusters 1, 2, 5: *Cd3e*, *Cd3g*), proliferative T cells (cluster 5: *Top2a*), NK cells (cluster 6: *Ncr1*, *Klrk1*), neutrophils (cluster 4: *Hdc*, *G0s2*), macrophages (cluster 3: *Adgre1*, *Fcgr1*), and DCs (cluster 7: *Flt3*, *Clec10a*) (Figure 6, A and B, and Supplemental Table 1). The type II receptor is expressed primarily on neutrophils and macrophages (Supplemental Figure 5A); accordingly, we observed unique neutrophil and macrophage clusters composed primarily of 4 dpi cells from rIL-13–treated animals (Supplemental Figure 5B).

We were specifically interested in defining macrophage phenotypes, so we reclustered original clusters 3 and 7, since cells from both clusters express macrophage markers (*Adgre1*, *Fcgr1*, *Mafb*) and were negative or low for neutrophil markers (*Hdc*, *Csf3r*, *G0s2*) (Figure 6C). After reclustering, we identified 5 subclusters (Figure 6D and Supplemental Figure 5C [Mac0-4]) 2 of which were identified as primarily DCs (*Flt3*, *Clec10a* [Mac2, Mac4]) or plasmacytoid DCs (*Siglech* [Mac4; pDCs]) (Supplemental Figure 5D and Supplemental Table 2). Of the macrophage clusters (Mac0, Mac1, and Mac3), Mac0 was almost entirely populated by 7 dpi macrophages, while cells from both time points and all treatment groups populated Mac1 (Figure 6, E and F). Interestingly, Mac3 was almost exclusively (~98%) composed of macrophages from rIL-13–treated mice at 4 dpi (Figure 6, E and F), indicating that this is a unique macrophage population appearing only in response to rIL-13. We performed Ingenuity Pathway Analysis (IPA) of up- and downregulated marker genes enriched in Mac0, Mac1, and Mac3 (Supplemental Figure 5E and Supplemental Table 3). While all clusters displayed distinct pathway enrichment, Mac3 macrophages strongly downregulate genes associated with proinflammatory processes (Pathogen Induced Cytokine Storm, Hypercytokinemia in Influenza); upregulated genes were associated with the antiinflammatory pathway IL-10. This analysis suggests an antiinflammatory macrophage phenotype induced by rIL-13. Macrophage phenotypes at 4 and 7 dpi differ tremendously. Considering that cells from Mac3 were derived almost entirely from 4 dpi hearts and that we found very few differentially expressed genes between rIL-13 and PBS macrophages at 7 dpi, we next focused our analysis on enriched genes in Mac1 or Mac3, focusing on cells only from the 4 dpi time point. This comparison allowed us to better elucidate differences between treatments at 4 dpi only. Pathway analysis revealed strong upregulation of proinflammatory pathways in Mac1(4dpi) cluster (interferon signaling, pathogen induced cytokine storm, hypercytokinemia in influenza; Figure 6H and Supplemental Table 4). The most strongly enriched genes populating these pathways included *Irf7*, *Isg15*, and Oas family members (*Oas3*, *Oas1a*, *Oasl2*; Figure 6G), all of which are induced in response to IFN (40). When comparing all cells from 4 and 7 dpi time points, these IFN-related genes were abundantly expressed in Mac1 clusters and showed moderate expression in Mac0 (primarily 7 dpi), but they were almost undetectable in Mac3 (Figure 6I). Thus, Mac1 macrophages represent a proinflammatory cluster that strongly contrasts with the antiinflammatory profile observed in Mac3 cells.

When analyzing only 4 dpi cells, Mac3 macrophages show comparatively strong expression of phagosome formation pathway genes (Figure 6H and Supplemental Table 4). Indeed, phagosome-related genes (*Clec4d*, *Frp2*) were strongly and specifically expressed in Mac3 when all cells, 4 and 7 dpi, were analyzed together (Figure 6J), suggesting rIL-13 induced a phagocytic macrophage phenotype that is not present at 4 dpi or 7 dpi under normal conditions. Similarly, rIL-13 strongly induced matrix remodeling (*Mmp8* and *Mmp19*) genes, which were nearly exclusively expressed by Mac3 cells when all cells were considered (Figure 6J). Collectively, pathway and gene expression analysis revealed a completely unique subpopulation of macrophages present at 4 dpi in response to rIL-13 administration. Based on expression analysis, these cells appear to be antiinflammatory and phagocytic, and they appear to secrete proteins that digest and remodel extracellular matrix.

### Identification and characterization of rIL-13–induced macrophages in vivo.

**Figure 7:**
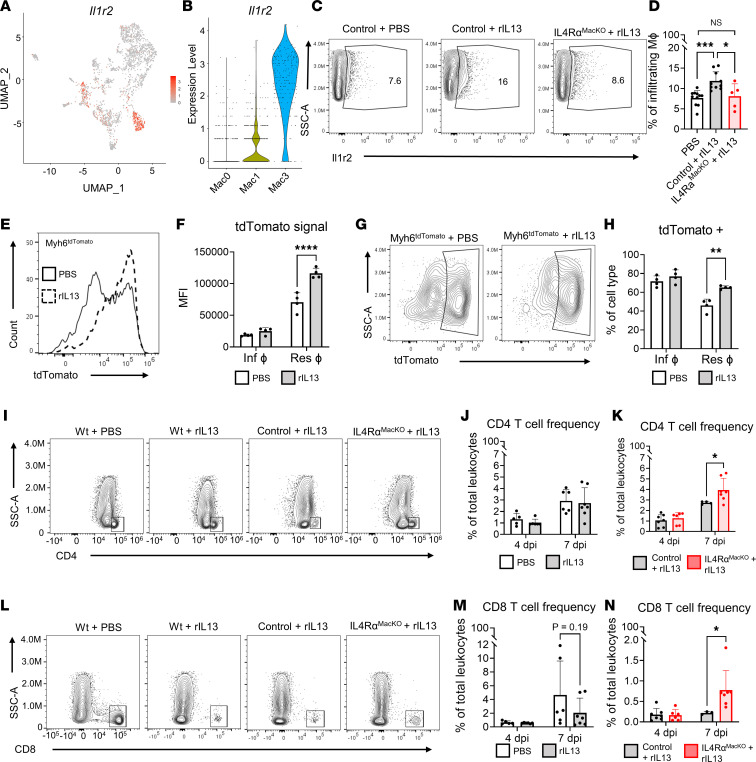
IL-13/IL-4Rα reactivation in macrophages induces IL-1R2 expression in vivo. (**A** and **B**) Feature plot of IL-1r2 expression in macrophages and violin plots of IL-1r2 expression in Mac0, Mac1, and Mac3 clusters. (**C**) Representative contour plots showing IL-1r2 expression in infiltrating cardiac macrophages at 4 dpi after MI. (**D**) Quantification of IL-1r2^+^ cardiac macrophage frequency at 4 dpi after MI. (**E**) Representative histograms showing tdTomato signal in cardiac resident macrophages from Myh6^tdTomato^ mice treated with PBS versus rIL-13 after MI. (**F**) Quantification of tdTomato mean fluorescence intensity (MFI) in cardiac macrophages from Myh6^tdTomato^ mice treated with PBS versus rIL-13 after MI. (**G** and **H**) Representative contour plots and quantification of tdTomato^+^ cardiac resident macrophages in Myh6^tdTomato^ mice treated with PBS versus rIL-13 after MI. (**I**) Representative contour plots showing cardiac CD4 T cell populations in WT mice treated with PBS versus rIL-13 and control and IL-4Rα^MacKO^ mice treated with rIL-13 after MI. (**J** and **K**) Quantification of cardiac CD4 T cells frequencies after MI in WT mice, and control and IL-4Rα^MacKO^ mice treated with rIL-13 after MI. (**L**) Representative contour plots showing cardiac CD8 T cell populations in WT mice treated with PBS versus rIL-13 and control and IL-4Rα^MacKO^ mice treated with rIL-13 after MI. (**M** and **N**) Quantification of cardiac CD8 T cell frequencies in WT mice, and control and IL-4Rα^MacKO^ mice treated with rIL-13 after MI. Data are shown as mean ± SD. Each data point represents 1 mouse. *P < 0.05, **P < 0.01, ***P < 0.001, ****P < 0.0001. Comparison by 1-way ANOVA and Tukey’s post hoc test in **D**. Treatment effect by 2-way repeated-measures ANOVA and Sidak’s post hoc comparison in **F** and **H**. Time effect by 2-way ANOVA and Sidak’s post hoc comparison in **J** and **M**. Interaction effect of time and genotype by 2-way ANOVA and Sidak’s post hoc comparison in **K** and **N**.

Our scRNA-Seq experiment identified a unique macrophage population observed only following rIL-13 administration. However, this experiment alone does not determine if this population is indeed responsible for proreparative phenotypes in vivo. We found that expression of *Il1r2*, which is a decoy receptor for IL-1 and therefore attenuates inflammation (41), was the most strongly enriched transmembrane receptor in Mac3 (Figure 7, A and B, and Supplemental Figure 5C). *Il1r2* was also strongly and significantly upregulated in macrophages from rIL-13 versus PBS-treated mice at 4 dpi, specifically (Figure 6G and Supplemental Table 5), suggesting that this surface receptor could serve as a faithful marker of proreparative macrophages in vivo. We developed a flow cytometry assay to detect this population in the heart following MI in mice. Indeed, at 4dpi in PBS-treated mice, we detected only about 5% of macrophages IL-1r2^+^, a negligible increase from levels detected in the fluorescence minus one (FMO) control. On the other hand, nearly 20% of both tissue-resident and infiltrating macrophages were IL-1r2^+^ following rIL-13 treatment (Supplemental Figure 6, A and B). Next, we tested if IL-1r2^+^ macrophages depended on IL-13 signaling to macrophages. We found that IL-1r2 frequency among macrophages of control and IL-4Rα^MacKO^ mice treated with PBS was similar (Supplemental Figure 6C). However, IL-1r2^+^ macrophages were enriched in the heart of control mice but not in the hearts of IL-4Rα^MacKO^ mice treated with rIL-13 (Figure 7, C and D). We also quantified frequency of CD206^+^ macrophages, since this receptor is widely used as a marker of M2-like macrophages and is reported to be induced by IL-4 or IL-13 (27, 42). CD206^+^ macrophages were increased in infiltrating macrophages following rIL-13 administration in adults; however, this marker was not elevated in tissue-resident macrophages (Supplemental Figure 6, D and E). In contrast to IL-1r2^+^, CD206^+^ macrophages were enriched in the heart of both control and IL-4Rα^MacKO^ mice treated with rIL-13 (Supplemental Figure 6, F and G), and this demonstrates the specificity of IL-1r2 as a marker of IL-13–induced proreparative macrophages. Interestingly, while this population of macrophages is found at very low levels in the adult heart after MI under normal conditions, ~10% of cardiac macrophages were IL-1r2^+^ in neonatal mice at 4 dpi (Supplemental Figure 6, H and I). Importantly, IL-4Rα^MacKO^ neonates had virtually no IL-1r2^+^ macrophages at 4 dpi, as levels were comparable with FMO–IL-1r2 control, indicating that cardiac IL-1r2^+^ macrophages are induced exclusively by IL-4Rα signaling. In neonates, ~35% of cardiac macrophages were CD206^+^ in control mice, similar to what we found in IL-4Rα^MacKO^ hearts at 4 dpi (Supplemental Figure 6, J and K). Thus, while stimuli besides IL-13/IL-4Rα signaling can promote CD206 expression on macrophages, IL-1R2^+^ macrophages represent what we consider to be a novel proreparative macrophage subtype specifically induced by IL-4Rα activation. Consistently with IL-4/IL-13 expression (Figure 2), IL-1R2^+^ macrophages are present in neonatal hearts but are nearly absent in adult hearts following injury.

Since our scRNA-Seq data suggest that IL-13–induced macrophages were highly phagocytic, we developed an assay to test macrophage phagocytic capacity in vivo. Following MI and daily rIL-13 or PBS injection in WT mice, *E. coli* bioparticles that become fluorescently green inside the acidic phagolysosome were administered by i.v. injection 2 hours prior to tissue harvest. We found that ~15% of macrophages were positive for *E. coli* bioparticle signal following rIL-13 administration, compared with ~3% in PBS-treated mice (Supplemental Figure 6, L and M). Macrophage phagocytosis of CM-derived debris is a protective mechanism that correlates with preserved function after cardiac injury (12, 20). Thus, we next assessed if IL-13–induced macrophages were specifically phagocytosing CM debris in the injured heart. Myh6^Cre^ mice were bred with the Rosa-26^tdTomato^ reporter line to generate mice, whereas CMs express tdTomato (Myh6^Cre^;Rosa-26^tdTomato^ [Myh6^tdTomato^]). In this model, the tdTomato signal in leukocytes is indicative of cell efferocytosis of CMs. Among leukocytes, macrophages primarily displayed a tdTomato signal (Supplemental Figure 6N) at 4 dpi, consistent with an efferocytotic phenotype of this cell type. rIL-13 administration to Myh6^tdTomato^ mice after MI resulted in higher tdTomato expression and higher frequency of tdTomato^+^ cardiac resident macrophages at 4 dpi (Figure 7, E–H), indicating that IL-13–induced macrophages are highly efferocytotic and have increase capacity to remove CM debris in the heart after MI.

Collectively, these results indicate that IL-13/IL-4Rα macrophage activation induces a unique macrophage cluster during the inflammatory phase after MI. These IL-13–induced macrophages are highly efferocytotic and remove more CM debris from the injured myocardium, have high expression of matrix remodeling genes, and can be identified by the expression of IL-1r2. Interestingly, IL-1r2^+^ macrophages are a completely distinct subpopulation that is independent from CD206^+^ M2-like macrophages.

### rIL-13 signaling to macrophages influences cardiac T cell frequency after MI.

Next, we assessed how IL-13 signaling to macrophages secondarily influences other immune cells in the heart post-MI. We quantified the frequencies of neutrophils and T cells in the postinfarct heart. Neutrophils were highly abundant in the heart at 4 dpi after MI, but their frequency among total leukocytes declined by 7 dpi (Supplemental Figure 6, O and P). rIL-13 administration increased neutrophil frequency at 7 dpi but not at 4 dpi (Supplemental Figure 6, O and P). However, neutrophil frequency at 7 dpi was not dependent on rIL-13 signaling to macrophages, since control and IL-4Rα^MacKO^ treated with rIL-13 showed no difference (Supplemental Figure 6Q).

T cells also have a major role in the injured heart, since CD4 Th17 cells accumulate in the heart and contribute to interstitial fibrosis and overall cardiac function deterioration (43). Furthermore, CD8 T cells infiltrate the infarcted heart and contribute to CM apoptosis through granzyme B release, resulting in additional CM apoptosis, exacerbated cardiac remodeling, and functional decline (44, 45). In WT mice, CD4 T cell frequency increased from 4 to 7 dpi, independently of rIL-13 administration (Figure 7, I and J). Interestingly, IL-4Rα^MacKO^ mice treated with rIL-13 had significantly increased CD4 T cell frequency at 7 dpi compared with control littermates (Figure 7, I and K). Along the same lines, CD8 T cell frequency increased from 4 to 7 dpi in WT mice, whereas rIL-13 treatment showed a trend toward suppressed CD8 frequency at 7 dpi (Figure 7, L and M). Similar to CD4 T cells, rIL-13 administration to IL-4Rα^MacKO^ exacerbated CD8 T cell infiltration at 7 dpi (Figure 7, L and N). These data indicate that macrophage IL-13/IL-4Rα activation has an ameliorating effect on cardiac infiltration of CD4 and CD8 T cells specifically in the context of rIL-13 administration.

## Discussion

The post-MI response in adult mammals differs greatly from that observed in organisms capable of cardiac regeneration such as neonatal mice and zebrafish (11, 46, 47). In nonregenerative scenarios, myocardial loss is nonreversible, since the initial injury leads to a strong inflammatory response, scar deposition, and fibrosis, which ultimately culminates in the development of HF. Regenerative organisms display a distinct and remarkable inflammatory response considered proreparative in nature. In neonatal mice, for example, the macrophage response is mediated primarily by expansion of cardiac resident macrophages as opposed to the monocytic infiltration seen in adults (1, 19). These macrophages display high angiogenic capacity, are weakly inflammatory, and lack intrinsic fibrosis-inducing properties like those observed in adults (11, 19, 23). The unique inflammatory profile, along with CM proliferative capacity, contributes to efficient regeneration of damaged myocardium in neonates (11, 46, 47). Thus, regenerative organisms are attractive models for identifying proreparative mechanisms that can be therapeutically applied to treat ischemic HF in adults.

Here, we leverage the neonatal mouse model of cardiac regeneration to identify a proreparative immune mechanism that we reactivate in adult stages to improve outcomes after MI. Our study, and recent publications from our group, elucidate a pleiotropic role for IL-13/IL-4Rα signaling in cardiac regeneration and repair (24, 25). In neonates, IL-4Rα expression in CMs and macrophages mediates distinct and complementary processes, both of which contribute to complete and functional cardiac regeneration. Namely, CM IL-4Rα deletion impairs CM cell cycle activity and results in increased scarring (25), while macrophage IL-4Rα deletion did not influence CM regeneration but was clearly required to prevent excessive scarring and to mediate functional recovery. There is currently a heightened interest in the role of CM cell cycle activation in the cardiac injury response (48), since proliferation of adult CMs is thought to be a promising strategy for the promotion of adult mammalian heart regeneration. While rIL-13 can enhance CM DNA synthesis in adults after MI, this response had no effect on functional recovery; therefore, our model segregates CM DNA synthesis and cell cycle activity from appreciable functional recovery phenotypes.

A growing body of literature has elucidated the profound influence that macrophages have on cardiac repair across developmental time points. While IL-13 is known to directly polarize macrophages, the phenotypic consequence of macrophage IL-13 signaling is variable across tissue types and disease conditions; it can be proreparative in some cases (22, 49) and, in other cases, can drive inflammation and fibrosis (50, 51). The role of IL-13 signaling to macrophages in the heart after MI has not been previously defined. By using a combinatorial approach of exogenous rIL-13 administration to cell type–specific genetic deletion models, we demonstrate, for the first time to our knowledge, that type II receptor signaling in macrophages clearly mediates functional recovery and prevents exacerbated scarring after MI in adult mice. While we mechanistically tested whether rIL-13 acts through CMs or macrophages in the adult to mediate post-MI phenotypes, our data suggest that rIL-13 also signals to yet another cardiac cell type, or cell types, to aggravate scarring — a phenotype only observed when IL-4Rα is deleted from macrophages. A similar case where IL-13 has disparate effects on neighboring cells has been demonstrated in the liver in the context of *Schistosoma* *mansoni* infection (28). In this scenario, IL-13 signals directly to biliary epithelial cells to induce cell proliferation and hyperplasia, and it independently signals to liver fibroblasts promoting liver fibrosis and eosinophilia. Thus, variable IL-13 signaling to these 2 cell types determined pathogenesis of liver fibrosis and biliary hyperplasia (28). In the heart, cell types such as neutrophils, fibroblasts, and endothelial cells express the IL-13 receptor (52) and could contribute to the aggravated phenotypes we observed when rIL-13 was administered to IL-4Rα^MacKO^ animals. Combinatorial cell-specific genetic deletion models targeting various cell types could be explored in future studies. These experiments are necessary for understanding therapeutic potential of activating IL-13 signaling after cardiac ischemic injury.

Macrophage IL-13/IL-4Rα stimulation resulted in enhanced capillary density after MI in neonatal mice but not in adult mice. This discrepancy may be due to IL-13/IL-4Rα signaling contributing to the angiogenic phenotype in cardiac resident macrophages, which overwhelmingly mediate cardiac regeneration in neonates (11, 19, 53). In the adult heart, the macrophage response is primarily derived from monocytes, which are less angiogenic than the neonatal counterparts (19) and could explain the discrepancy. Nevertheless, macrophage IL-13/IL-4Rα signaling mediated functional recovery after injury in both scenarios, suggesting that the angiogenic phenotype seen in neonates is correlational only and that other mechanisms such as enhanced phagocytosis or scar remodeling are behind this improvement.

Emerging literature has illustrated the central role that macrophages play during recovery after cardiac injury (1, 2, 4, 11), and recent transcriptomic analyses have identified multiple macrophage subpopulations with unique characteristics that differentially contribute to cardiac repair (3, 5, 6, 15). Seminal studies have validated broad macrophage classes by cell depletion studies (3, 6, 11), but the specific pathways promoting reparative cardiac macrophage profiles are relatively underexplored. rIL-13 signaling to macrophages induced a unique cluster of IL-1r2^+^ cells that express high levels of matrix remodeling genes, are highly efferocytotic, and remove higher amounts of CM debris in the injured myocardium. Macrophage efferocytosis of necrotic CM debris and apoptotic cells improves cardiac function after MI (12, 20, 42), and many studies have demonstrated that expression of MMPs increases after MI and contributes to scar removal (54). Thus, it is plausible that enhanced macrophage efferocytosis of necrotic CM debris and MMP secretion both contribute to improved outcomes after MI in our model. Surprisingly, expression of CD206, a classical M2 marker well described to be induced by IL-13/IL-4 signaling, was minimally influenced by rIL-13 administration in cardiac macrophages after MI, while IL-1R2 served as a faithful surface receptor labeling rIL-13–activated cardiac macrophages. IL-1r2 expression itself could also have a functional effect, since it is a decoy receptor for the proinflammatory cytokine, IL-1b (41), and thereby prevents IL-1b activity. IL-1R2 is elevated in patients after acute coronary syndrome and in mice after cardiac ischemia and reperfusion (I/R) injury (41). In the same studies, IL-1r2 genetic deletion resulted in aggravated response to I/R, while IL-1r2 overexpression prevented CM apoptosis (41).

IL-13/IL-4Rα macrophage signaling suppressed the frequencies of CD4 and CD8 T cells in the heart after MI. Taken together, these data suggest that systemic delivery of rIL-13 signals to other nonmacrophage cell types expressing the type II receptor, such as neutrophils, fibroblasts, endothelial cells, or CMs, which drive a secondary T cell response. However, rIL-13 signaling directly to macrophages ameliorates the infiltration of CD4 and CD8 T cells in the myocardium after MI, and exacerbated frequencies of CD4 and CD8 T cells in the myocardium of rIL-13–treated IL-4Rα^MacKO^ mice correlate with scar aggravation after MI. Importantly, decreased T cell frequencies could have a direct effect on heart function after MI, as these cells have been linked to disease progression in patients and in animal models. CD4 T cells are more abundant in biopsies from patients with advanced HF (43), which additionally had increased adherence to endothelial cells, suggesting an increased activation state (43). Along the same lines, CD8 T cells have a negative effect on cardiac function following MI. In one study, CD8 T cells were found to secrete granzyme B in the BZ myocardium after MI, aggravating CM apoptosis and functional decline in mice (44). In a more recent study, lymphatic endothelial cells were found to proliferate in the ischemic myocardium after MI and to express increased levels of the transcription factor Tbx1. This resulted in reduced number of CD8 T cells, which had a beneficial role in the heart after MI (45).

In summary, we demonstrate a central role for IL-13 signaling to macrophages in the post-MI injury response. By leveraging the neonatal mouse cardiac regeneration model, we identified a proreparative signaling axis, IL-13/IL-4Rα, that declines precipitously into adulthood. Our work exemplifies how re-reactivation of this pathway in cardiac macrophages in adults improves post-MI outcomes and implies that activation of IL-13 signaling to macrophages could be developed as a therapeutic strategy for treating ischemic HF in humans.

## Methods

Supplemental Methods are available online with this article.

### Statistics.

Statistical analysis was performed with GraphPad Prism 8.3 software. Statistical comparisons between 2 groups were analyzed using unpaired, 2-tailed Student’s *t* test or between 3 or more unrelated groups by 1-way ANOVA followed by Tukey’s multiple-comparison test. Statistical comparison between 2 groups subjected to 2 treatments or assessed at different time points was performed by 2-way ANOVA with repeated measures when appropriate (i.e., different time points of same individual or different cell types of same sample) followed by Sidak’s multiple comparison test. All data are presented as mean ± SD unless specified otherwise. Post-MI Kaplan Meier survival curves were analyzed using Mantel-Cox log-rank test comparison. *P* < 0.05 was considered significant throughout.

### Study approval.

All protocols and experiments in this study were approved by the local Animal Care and Use Committee of The Medical College of Wisconsin, an The Association for Assessment and Accreditation of Laboratory Animal Care–approved (AAALAC-approved) facility.

### Data availability.

The data presented in this publication have been deposited in NCBI’s Gene Expression Omnibus (GEO and are accessible through GEO Series accession no. GSE231707. Our submission is MINSEQE compliant. Values for all data points in graphs are reported in the Supporting Data Values file. 

## Author contributions

SAA, SJP, MAF, CWM, MCK, VAA, SLB, MP, and ABN performed experiments and collected and analyzed data. SAA and CCO wrote most of the manuscript. SAA, SJP, MP, YGC, and CCO conceptualized and designed experiments. CWL performed data analysis. All authors edited the manuscript. CCO obtained funding to support this project.

## Supplementary Material

Supplemental data

Supplemental table 1

Supplemental table 2

Supplemental table 3

Supplemental table 4

Supplemental table 5

Supplemental table 6

Supplemental table 7

Supplemental table 8

Supporting data values

## Version 1 12/05/2023

In-Press Preview

## Version 2 01/23/2024

Electronic publication
