# Supplementary material for: IL-13 promotes functional recovery after myocardial infarction via direct signaling to macrophages
Source: JCI Insight. 2024 Jan 23;9(2):e172702. doi: 10.1172/jci.insight.172702 (PMC10906228; doi:10.1172/jci.insight.172702)
Supplement: Supplemental data [file jciinsight-9-172702-s174.pdf]

DATA SUPPLEMENT TO:

Exogenous Interleukin 13 Promotes Functional Recovery after Myocardial Infarction via Direct Signaling to Macrophages

Santiago Alvarez-Argote[1,5], Samantha J. Paddock[1,5], Michael A. Flinn[1,5], Caelan W. Moreno[1,5], Makenna C. Knas[1,5], Victor Alencar Almeida[1,5], Sydney L. Buday[1,2,5], Amirala Bakhshian Nik[1,5], Michaela Patterson[2,5], Yi-Guang Chen[3], Chien-Wei Lin[4], and Caitlin C. O'Meara[1,5]

MATERIALS & METHODS

Animals

Mice were housed in the Biomedical Resource Center at the Medical College of Wisconsin, an AAALAC-approved facility. For all *in vivo* animal experiments, data from male and female mice were pooled. IL4Rα-floxed (IL4Rα$^{f/f}$) mice were received as a gift from Dr. Frank Brombacher (1) from the University of Cape Town, Cape Town, South Africa and backcrossed to C57BL/6J background (Jackson laboratories 000664). These mice were crossed with B6J.B6N(Cg) Cx3cr1tm1.1(cre)Jung/J (Cx3cr1^{Cre}) mice (Jackson Laboratories 025524) or B6.FVB-Tg(Myh6-cre)1282Mds/J (Myh6^{Cre}) mice (Jackson Laboratories 011038) to generate IL4Rα^{MacKO} and IL4Rα$^{CM\text{-}KO}$ mice and littermate controls. C.129S4(B6)-Il13tm1(YFP/cre)Lky/J mice (Jackson laboratories 017353) knock-in (KI) reporter mice were backcrossed to BALB/c mice (Jackson Laboratory 000651) to generate IL13YFP$^{ki/wt}$ (YFP$^+$) and IL13YFP$^{wt/wt}$ (YFP$^-$) littermates. C.129-Il4tm1Lky/J KI reporter mice (Jackson laboratories 004190), were backcrossed to BALB/c mice (Jackson Laboratory 000651) to generate IL4GFP$^{ki/wt}$ (GFP$^+$) and IL4GFP$^{wt/wt}$ (GFP$^-$) littermates. Rosa26-tdTomato (B6.Cg-Gt(ROSA)26Sortm9(CAG-tdTomato)Hze/J), were obtained from Jackson laboratories (stock number 007909) and crossed with Myh6^{Cre} mice to generate Myh6^{tdTomato} mice and Cre negative (Myh6^{wt}) littermate controls.

Neonatal Myocardial Infarction (MI) Model

MI in postnatal day 1 (P1) neonatal mice was performed as previously described (2, 3). Briefly, P1 mice were anesthetized on ice and thoracotomy was performed. The chest was retracted to expose the heart, manual pressure was applied on the right hemithorax to protrude the heart, and the left anterior descending (LAD) coronary artery was

visualized using a surgical microscope and ligated at approximately 2-3 mm below the left atrial appendage using polypropylene 7-0 (PROLENE, Ethicon EP8734H) suture. The rib incision was also sutured with PROLENE 7-0, and skin glue was used to close the skin incision. Pups recovered under a heat lamp until responsive and then were returned to their mother.

Adult MI Model

MI was surgically induced by permanent LAD ligation in adult (8- to 9-week-old) mice. We used the "rapid" surgical method (4). Briefly, mice were anesthetized using 2%/98% isoflurane/O2 gas mix at 2 Lt per minute, chest hair was removed using Nair hair removal (Church & Dwight), and skin was cleaned and sanitized using iodopovidone and ethanol. Skin on the left hemithorax was incised using surgical scissors and the 4th intercostal space was bluntly dissected to create an opening into the thoracic cavity. Manual pressure was applied on the right hemithorax to protrude the heart through the opening. The LAD was directly visualized and ligated using PROLENE 6-0. The heart was allowed to retract into the thorax, the pneumothorax was minimized by applying manual pressure on the left hemithorax and skin was sutured with PROLENE 4-0. Mice were injected subcutaneously with slow-release buprenorphine 0.5 mg/mL at a dose of 4 mL/kg of body weight and 300 µL of 0.9% saline solution after surgery and let to recover under warm light and on a heating pad. One day following MI, mice were administered meloxicam oral solution 1.5 mg/mL at a dose of 7.5 mg/kg.

Bromodeoxyuridine (BrdU) and 5-Ethynyl-2-deoxyuridine (EdU) administration

BrdU (Sigma-Aldrich B5002) was dissolved in dimethyl sulfoxide (DMSO) at a concentration of 20 mg/mL. Neonatal mice received subcutaneous injections of BrdU

solution at 5 and 7 days post injury (dpi) at a dose of 100 mg/kg. EdU (Invitrogen A10044) was dissolved in DMSO and further diluted in phosphate buffered saline (PBS) to a working concentration of 4 mg/mL, adult mice received daily intraperitoneal (IP) injections from 4 to 10 dpi at a dose of 10 mg/kg.

Recombinant murine Interleukin 13 (rIL13) administration

Murine rIL13 (Preprotech, 210-13) was diluted in 0.1% bovine serum albumin (BSA), serving as a carrier protein, in PBS to a final concentration of 4 ng/µL. Adult mice were administered rIL13 at a dose of 10 µg/kg by daily intraperitoneal injections from days 2 to 10 post-MI and every other day from days 12 to 28 post-MI. Vehicle control littermate mice were administered 0.1% BSA/PBS following the same injection protocol as rIL13 treated animals. Previously, our laboratory characterized the plasma concentration of IL13 achieved with this regimen (5). Briefly, in adult mice administration of 100 ng of rIL13 resulted in peak plasma concentration of ~350 µg/mL at 4 hours after i.p. injection, with a half-life of approximately 12 hours. Thus, i.p. injections of rIL13 at a dose of 10 µg/kg every 24 hours is sufficient to saturate IL13 receptors systemically for the course of the injections.

pHrodo green E. coli BioParticles Conjugate injection

pHrodo green bioparticles (Invitrogen P35366) was reconstituted in sterile PBS to a final concentration of 2 mg/mL. Adult mice were anesthetized with 1.5%/98.5% isoflurane/O_2 mix at 1 Lt/minute flow rate and received pHrodo green bioparticles IV injections at a dose of 10 mg/kg in the tail vein 2 hours before euthanasia at 4 dpi after MI. One PBS treated mouse didn’t receive pHrodo injection so that it was considered the FMO-pHrodo green control.

Echocardiography

Mice were anesthetized with 1.5%/98.5% isoflurane/O_2 mix at 1 Lt/minute flow rate for echocardiography at 21 days after MI for P1 experiments and at days -1 (baseline), 3, 14 and 28 dpi for adult experiments. Echocardiography was performed using a VEVO 3100 (FUJIFILM; VisualSonics) echography machine equipped with a VEVO3100 imaging software and using the MX550D probe. The cardiac apex was aligned to the aorta to obtain longitudinal B-mode images of the left ventricle (LV). The LV circumference was outlined, and the area quantified during systole and diastole were used to calculate percent ejection fraction (%EF) and cardiac volumes. Then, the MX550D probe was turned 90º to obtain perpendicular and cross-sectional images of the left ventricle at the mid-papillary level. M-mode images were used to measure LV internal diameters during systole and diastole which were used to calculate percent fractional shortening (%FS). Images were analyzed with VEVO LAB software (FUJIFILM; VisualSonics). Delta FS (ΔFS 28 vs. 3 dpi) was calculated using the following formula: ((28 dpi %FS - 3 dpi %FS) / (baseline %FS — 3 dpi %FS)) x 100.

Euthanasia

For neonatal experiments mice were euthanized at 21 dpi, and for adult experiments mice were euthanized at day 28 dpi. Mice were deeply anesthetized with 3%/97% isoflurane to O2 mix at 2 Lt/minute flow rate until euthanized. Wide thoracotomy was performed, and the right atrium was nicked to allow blood to be cleared from the heart. For flow cytometry and cell sorting experiments, hearts were flushed with 3 mL of ice-cold PBS. For immunohistochemistry experiments, hearts were flushed with 3 mL of fresh 500 mM potassium chloride (KCl).

Heart fixation and processing

For histological experiments, hearts were placed in 4% paraformaldehyde (PFA) for 24 hours, then transferred to 70% ethanol for at least 48 hours, and subsequently embedded in paraffin blocks. Hearts were paraffin embedded in short-axis orientation to obtain “2-chamber view” tissue sections. Tissue sections of 4μm thickness were collected every 500 μm starting at the apex.

Immunohistochemistry

Formalin fixed and paraffin embedded tissue sections were deparaffinized with xylenes and rehydrated with 100%, 95%, and 75% ethanol, followed by water rehydration. Antigen retrieval was performed with pH 6.0 citrate buffer (Sigma-Aldrich C9999) in a water bath at sub-boiling temperature for 30 minutes. Tissue sections were blocked with 5% normal donkey serum (Sigma Aldrich D9663) and 1% BSA (Sigma Aldrich A3294) in 0.1% Triton X (Sigma Aldrich X100) in PBS (PBS-T). Primary antibody incubation was performed at 4˚C overnight, and secondary antibody incubation was performed for one hour at room temperature. 0.03% Sudan black in 70% EtOH was used to reduce background fluorescence. A list of primary and secondary antibodies can be found in **Table S8**. Fluorescent images were taken using an Eclipse Ni-E microscope (Nikon) and Nikon Digital Sight DS-Fi1 camera.

CD31 staining was counterstained with Wheat germ agglutinin (WGA) and DAPI (Biolegend 422801, 1μg/5mL) to calculate capillaries per CM. For this metric, two images of the left myocardium adjacent to the scar (border zone, BZ) and two images of the left myocardium remote to the scar (remote zone, RZ) were collected using a 40X objective and at a level of 1200 μm above apex for P1 mouse experiments and 1500 μm above

apex for adult mouse experiments. Sections that contained mostly myocardium fibers in cross-sectional orientation were selected. WGA staining was used for CM cross sectional area measurement. Quantifications were performed on Image J using an algorithm that normalized pixels to μm^2, applied the 'Phansalkar' local thresholding at a 15 radius setting, converted positive fluorescent signal and negative background into a binary format, and quantified number of particles per field and cross-sectional area of each particle. This analysis was applied to CD31 and WGA staining to obtain capillary and CMs information, respectively. The number of capillaries was normalized to the number of CMs (i.e., capillary density).

For BrdU/NKX2.5 and PCNA/Mef2 staining, two images of the remote myocardium at 500 μm above apex were taken. These metrics were assessed in 8 dpi mice after P1 MI, images were taken using the Ni-E microscope, and analyzed with image J manually by a blinded investigator. For EdU/Troponin (cTnnT) staining, Alexa fluor (AF) 488 ClickIT kit was used to stain for EdU incorporation, and AF555 Goat anti-mouse (**Table S8**) was used to detect cTnnT. Four images in the BZ and eight images in the RZ at 20X magnification were taken at 1000, 1500 and 2500 μm above apex were taken. We calculated an average for the BZ and for the RZ per mouse.

Scar assessment

Tissue sections were deparaffinized with xylenes and rehydrated with 100%, 95%, and 75% ethanol, followed by staining with Gömöri's trichrome. Images were scanned at a resolution of 4000 dots per inch (approximately 4X) using the super COOLSCAN 9000 (Nikon). Scar area was quantified in ImageJ using the color thresholding tool to outline the scar (blue) and viable myocardium (pink/purple). Scars were measured as areas in

mm^2 at multiple levels from apex through mid-papillary, and the summation of these areas is reported as absolute area in mm^2. To assess MI length we used the semi-automated MIQuant method (6).

Flow Cytometry

For flow cytometry and fluorescent cell sorting, hearts were digested to obtain single cell suspensions. Hearts were collected after PBS flushing, minced on a Moriah spoon, and resuspended in a mix of Hanks balanced salt solution (HBSS) with Calcium (Ca^{++}) and Magnesium (Mg^{++}) (Gibco 14025092; 1 mL per neonatal heart and 2 mL per adult heart), 3% fetal bovine serum (FBS; Sigma Aldrich F2442), Collagenase type II (Gibco 17101015; 250 units (U) per neonatal heart and 450 U per adult heart) and DNAse type I (Millipore Sigma D5025; 50 U per neonatal heart and 100 U per adult heart). Enzymatic digestion was stopped with 1mM Ethylenediaminetetraacetic acid (EDTA; Sigma Aldrich 03690) in HBSS without Ca^{++} and Mg^{++} (Gibco 14175095). Red blood cells were lysed with ammonium-chloride-potassium (ACK) lysis buffer (Gibco A1049201) and resuspended in 1% FBS, 0.1% NaN3, 1 mM EDTA in HBSS without Ca^{++} and Mg^{++} (FACS buffer) at a concentration of 1 million cells per 100 µl of FACS buffer. For flow cytometry, 1 million cells were used for subsequent staining, and for FACS, the whole sample was used for staining. Prior to staining, cells were incubated in Fc blocking solution (anti-mouse CD16/32, BD Biosciences 553141) to prevent unspecific Fc antibody binding.

Single cell suspensions were stained for live leukocytes, macrophages, T cells, B cells, ILC2, GFP/IL4 and IL4Rα receptor using the antibodies listed in **Table S8**. YFP/IL13 was assessed with the anti-GFP antibody. Flow cytometry was performed on hearts from uninjured mice or 4-, or 7- dpi using the 5-lasers Aurora Cytek spectral flow cytometer,

equipped with spectroFlo software (Cytek Biosciences). Flow cytometry to detect IL4Rα in neonatal mice was performed using the LSR Fortessa X20 cytometer (BD biosciences). Cells from adult spleens were stained for CD4 or CD8-fluorophore to obtain single color controls (**Table S8**). Fluorescent minus one (FMO) controls were used to assess IL4Rα, IL1R2 and CD206 staining. Cells from IL13YFP$^{wt/wt}$ or IL4GFP$^{wt/wt}$ animals were stained with anti-GFP antibody as a negative control for IL13YFP$^{ki/wt}$ or IL4GFP$^{ki/wt}$, respectively. To assess tdTomato signal in Myh6^{tdTomato} and Cre negative (Myh6^{wt}) littermates, 8- to 9-week-old mice were subjected to MI, treated with rIL13 or PBS, euthanized at 4 dpi and their hearts were digested as described above. TdTomato was assessed in cardiac macrophages and other immune cell types. Data was analyzed using FLOWJO software (BD biosciences), specific cell populations are reported as percentage of total live cells or percentage or parental population. IL4Rα-APC, GFP/YFP-AF488 and tdTomato are reported as mean fluorescent intensity (MFI) and percentage of parental populations (e.g., tdTomato/IL4Rα$^+$ resident macrophages or GFP$^+$ ILC2).

Single cell RNA sequencing (scRNAseq)

To evaluate the phenotypes of cardiac macrophages we collected cardiac leukocytes for scRNAseq at 4 and 7 dpi following MI and daily PBS or rIL13 injection in adult C57BL/6J mice. At 4 or 7 dpi, two heart ventricles per group were digested and pooled to obtain a single cell suspension following the same protocol for flow cytometry. Briefly, hearts were perfused with 3 mL of cold PBS, atria were removed, and ventricles were extracted, minced, and digested in a collagen type II, DNAse type I and HBSS with Ca^{++} and Mg^{++} solution. Following digestion, ventricular tissue was resuspended in 1mM EDTA HBSS and filtered through a 70 μm cell strainer to remove large cells and undigested

tissue. Erythrocytes were removed using ACK lysis buffer. Cells were counted using the LUNA^{TM} automated cell counter and resuspended to a concentration of one million cells per 100 µL of FACS buffer. Samples were stained for live leukocytes with an anti-CD45 antibody (**Table S8**) for 45 minutes, followed by incubation in 500 µL of DAPI (Biolegend 422801, 1:10,000) in FACS buffer for 5 minutes. Finally, samples were washed with 1 mL of FACS buffer, resuspended in 1 mL of FACS buffer and filtered through a 40 µm cell strainer just before sorting. Samples were sorted using BD FACS Melody Cell Sorter (BD Biosciences) and collected in FACS buffer.

Single cell capture, cDNA synthesis, barcoding, and library preparation was performed using the 10x Chromium system using V3.1 chemistry according to the manufacturer's recommendation (10x Genomics). Each sample was loaded onto a single lane of a Chromium Next GEM chip G with the goal of collecting 3,000 cells per sample. Cells were captured in single GEMs and cDNA was synthesized then amplified using 12 amplification cycles, followed by library construction per manufacturer's protocol. An i7 multiplex single index kit was used to generate the libraries over 14 cycles of sample index PCR. Fragment size of cDNA and libraries was assessed using Agilent's 5200 Fragment Analyzer System. Libraries were sequenced at the Linda T. and John A. Mellowes Center for Genomic Sciences and Precision Medicine of the Medical College of Wisconsin on a NovaSeq 6000 using one S4 flow cell with 2X150nt reads.

scRNAseq data analysis

Sequencing data were processed using 10x Genomics Cell Ranger 6.0.0 software for demultiplexing and generating cell barcode and gene count matrices for each sample. Four samples (two time points and two administrations) were merged and analyzed using

R package Seurat V4.3.0 (7). Cells with expressed number of genes less than 200 or greater than 5000 or percent of mitochondrial genes greater than 20% were filtered due to concerns of poor quality or doublets, resulting in 13,126 cells for downstream analysis. Data were normalized and scaled using function SCTransform (8) to adjust for different library sizes between cells and the variation due to percent of mitochondrial genes was further removed using non-regularized linear regression. The top 3,000 highest variable genes were selected and used for principal component analysis (PCA) to denoise and reduce the dimension of the data. Shared nearest-neighbor graph (SNN) of each cell was constructed based on the Euclidean distance using the first 30 principal components (PCs). Cell clusters were identified by a SNN modularity optimization-based clustering algorithm (Louvain-Jaccard graph-based method). The first 30 PCs were also used as input for Uniform Manifold Approximation and Projection (UMAP) dimension reduction method to visualize the data in two dimensions. Cluster marker genes were identified by function FindAllMarker, which performed differentially expressed (DE) gene analysis between two groups of cells using the Wilcoxon test. Dimension reduction plots, violin plots, feature plots and heatmaps were made by functions DimPlot, VlnPlot, FeaturePlot, and DoHeatmap respectively. Re-cluster analysis was done by first retrieving the cells in cluster 3 and 7 and then followed by the same pre-processing procedures (SCTransform normalization and PCA denoising) and downstream analysis (clustering analysis and marker gene DE analysis). Identification of cluster cells types was done by importing cluster marker genes into panglaoDB (9) and pathway analysis was performed using Ingenuity Pathway Analysis (Qiagen).

Figures S1

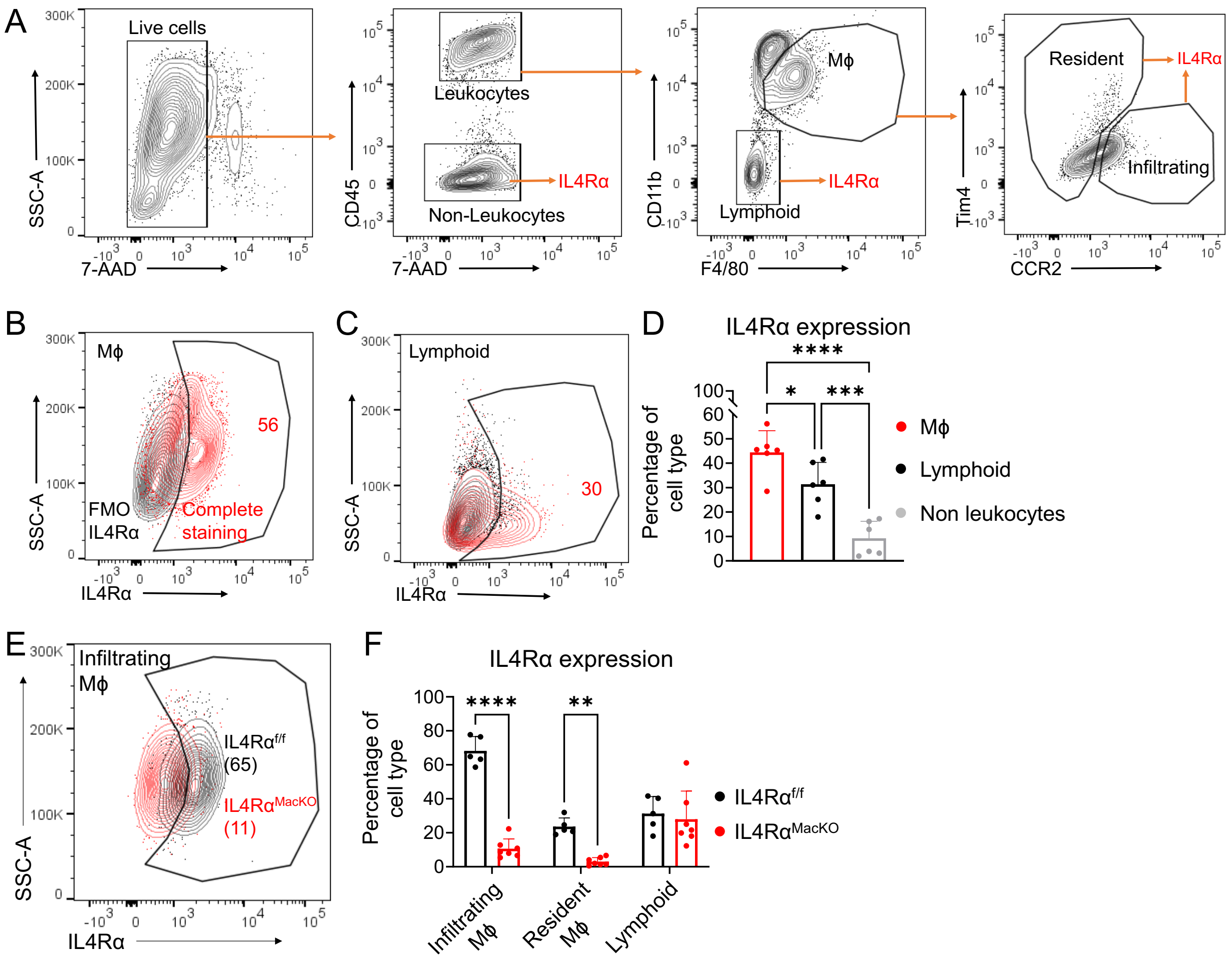

A. Contour plots showing gating strategy to assess IL4Rα expression in infiltrating and resident macrophages (Mφ), lymphoid cells and non-leukocytes. **B.** Representative contour plots showing IL4Rα expression in infiltrating macrophages and lymphoid cells (**C**) in IL4Rα$^{f/f}$ hearts at 4 days post injury (dpi) after myocardial infarction (MI) at post-natal day 1 (P1). **D.** Quantification of IL4Rα positive cells at 4 dpi after P1 MI. **E.** Representative contour plots showing IL4Rα expression in infiltrating macrophages of IL4Rα$^{f/f}$ vs IL4Rα^{MacKO} mice at 4 dpi after P1 MI. **F.** Quantification of IL4Rα expression as percentage of cell type for IL4Rα$^{f/f}$ vs IL4Rα^{MacKO} hearts at 4 dpi after P1 MI. Data is shown as mead ±SD. Each data point represents one mouse. *P<0.05, **P<0.01,

***P<0.001 and ****P<0.0001. Comparison by one way (1w) analysis of variance (ANOVA) and Tukey's post-hoc test in D. Interaction effect between cell type and genotype by 2w ANOVA and Sidak's post-hoc comparison for resident and infiltrating macrophages but not lymphoid cells between genotypes in F.

Figure S2

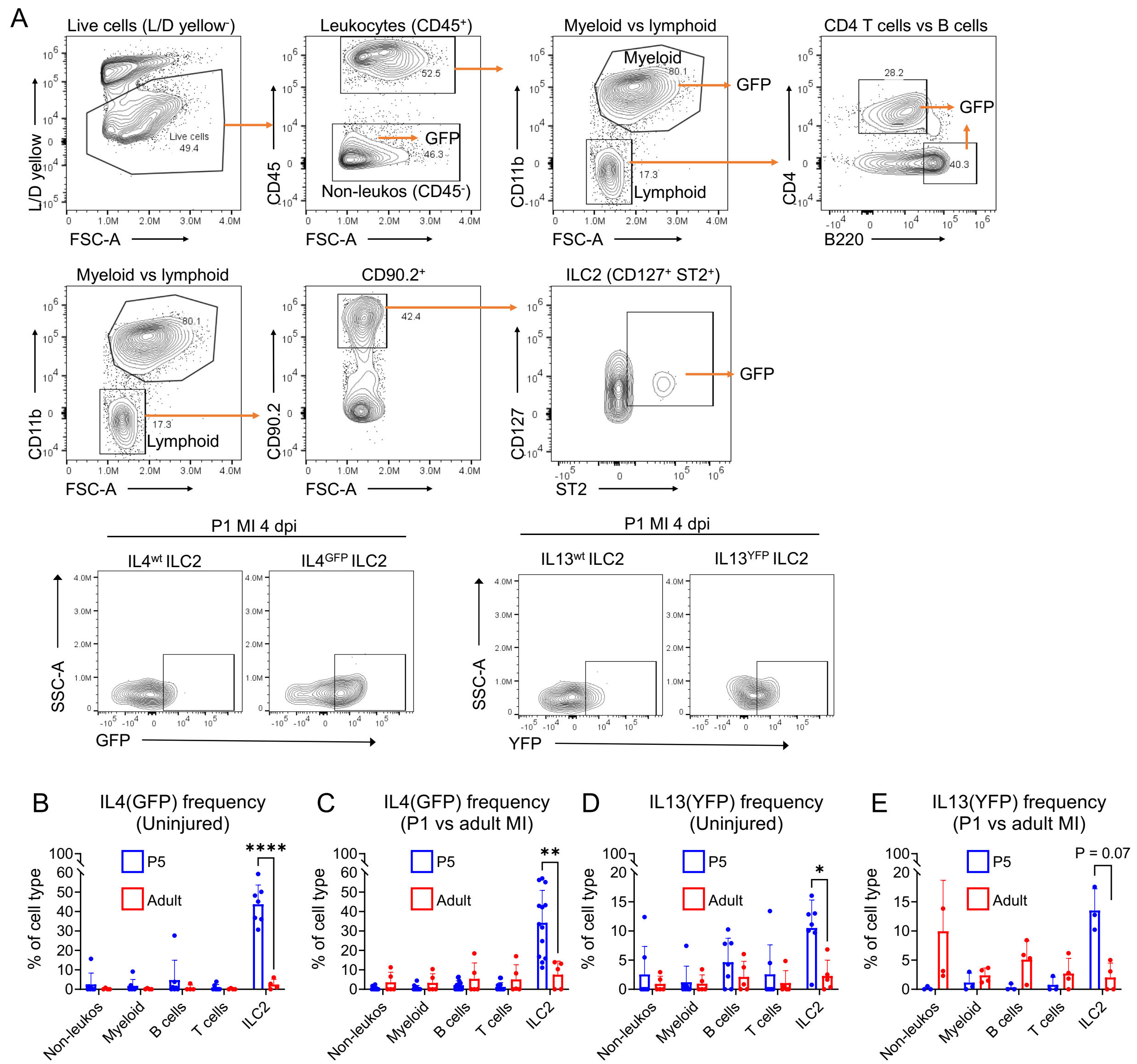

A. Gating strategy to assess IL4/GFP and IL13/YFP expression on type 2 innate lymphoid cells (ILC2), myeloid cells, B cells, T cells and non-leukocytes in P6 and 8-week-old (adult) mice. **B.** Quantifications of GFP/IL4 expression among multiple cell types in neonatal versus adult mice in hearts of uninjured and (**C**) infarcted mice. **D.** Quantification of YFP/IL13 expression among multiple cell types in neonatal versus adult mice in hearts of uninjured and (**E**) infarcted mice. Data are shown as mean ±SD. Each data point

represents one mouse. *P<0.05, **P<0.01 and ****P<0.0001. Interaction effect of cell type and age by 2w RM ANOVA and Sidak's post-hoc comparison in B-E.

Figure S3

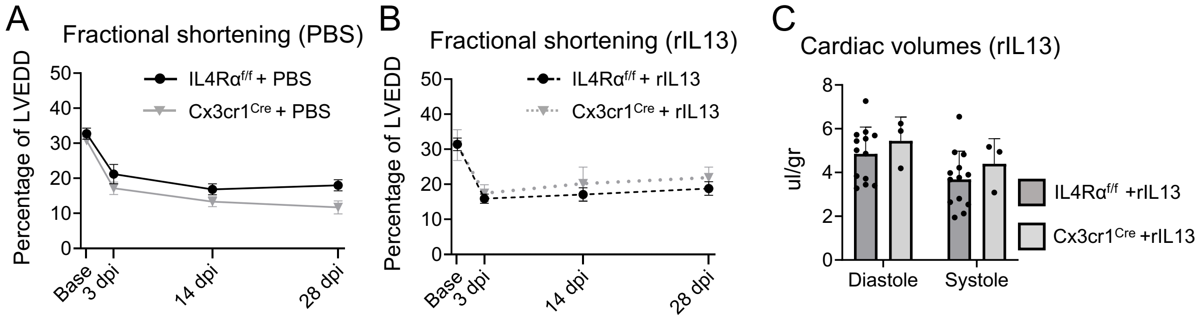

A. Fractional shortening (FS) at baseline (base), 3, 14 and 28 dpi in IL4Rα$^{f/f}$ and Cx3Cr1^{Cre} mice injected with PBS. IL4Rα$^{f/f}$ (N = 14), Cx3Cr1^{Cre} (N = 3). **B.** FS at baseline (base), 3, 14 and 28 dpi in IL4Rα$^{f/f}$ and Cx3Cr1^{Cre} mice injected with recombinant IL13 (rIL13). IL4Rα$^{f/f}$ (N = 11), Cx3Cr1^{Cre} (N = 3). **C.** Quantification of cardiac volumes at 28 dpi after MI and rIL13 administration for IL4Rα$^{f/f}$ and Cx3Cr1^{Cre} mice. 2w RM ANOVA in A, B and C.

Figure S4

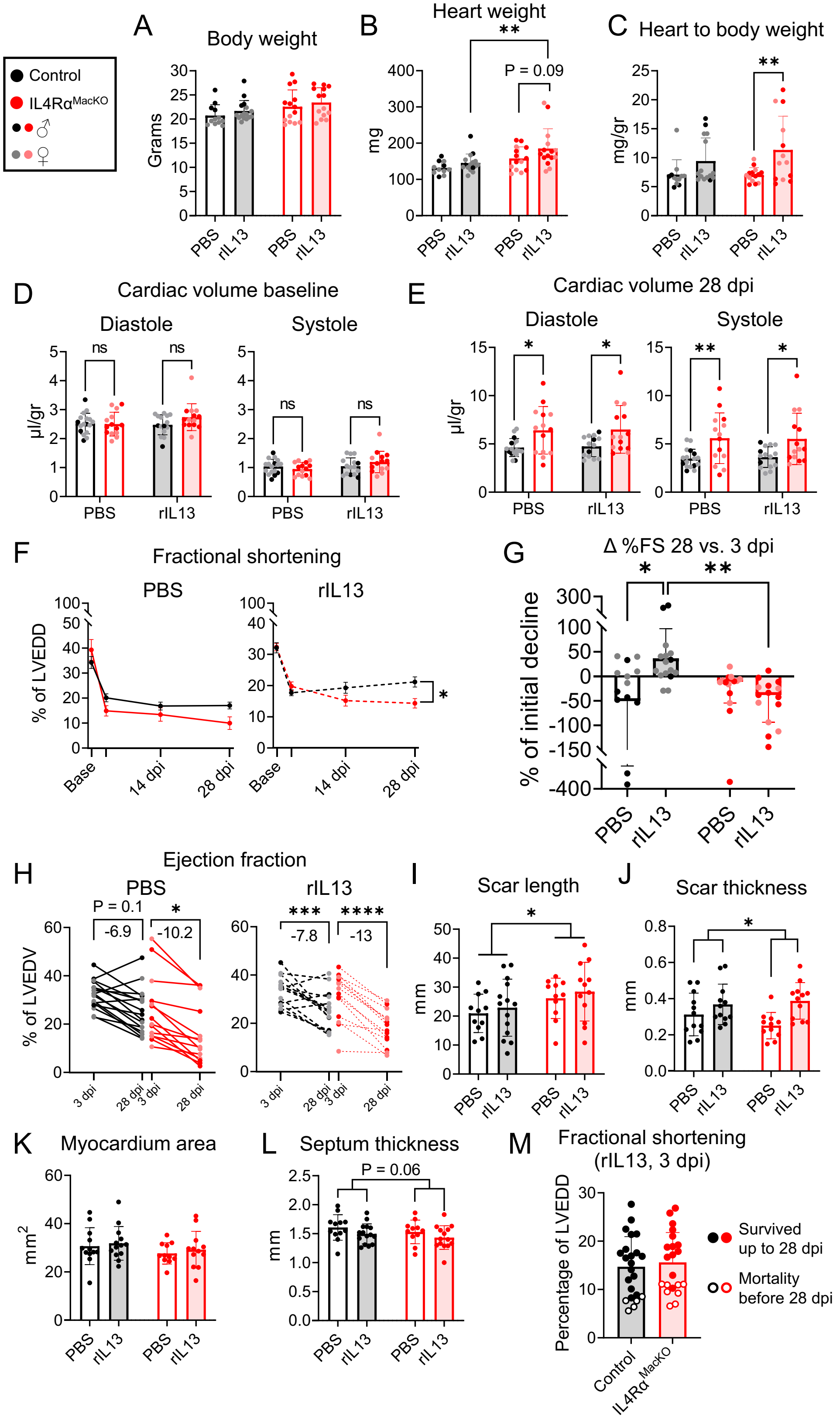

Control
IL4Rα MacKO
♂
♀
A
Body weight
Grams
PBS
rIL13
B
Heart weight
mg
P = 0.09
**
C
Heart to body weight
mg/gr
**
D
Cardiac volume baseline
Diastole
Systole
μl/gr
ns
E
Cardiac volume 28 dpi
Diastole
Systole
μl/gr
F
Fractional shortening
PBS
rIL13
% of LVEDD
Base
14 dpi
28 dpi
G
Δ %FS 28 vs. 3 dpi
% of initial decline
H
Ejection fraction
PBS
rIL13
% of LVEDV
P = 0.1
-6.9
-10.2
-7.8
-13
3 dpi
28 dpi
I
Scar length
mm
J
Scar thickness
mm
K
Myocardium area
mm2
L
Septum thickness
mm
P = 0.06
M
Fractional shortening (rIL13, 3 dpi)
Percentage of LVEDD
Control
IL4Rα MacKO
Survived up to 28 dpi
Mortality before 28 dpi

Group colors in the inset are used throughout entire figure. **A.** Body weight, (**B**) heart weight, and (**C**) heart to body weight at 28 dpi. **D.** Cardiac volume during diastole and systole at baseline and (**E**) at 28 dpi. **F. %**FS at baseline, 3, 14 and 28 dpi after MI and PBS vs. rIL13 administration. Control + PBS (N = 17), L4Rα^{MacKO} + PBS (N = 12), Control + rIL13 (N = 14), L4Rα^{MacKO} + rIL13 (N = 15). **G.** Delta %FS (FS at 28 dpi – FS at 3 dpi) /(FS at baseline – FS at 3 dpi). **H.** Ejection fraction (%EF) at 3 and 28 dpi after MI and PBS vs. rIL13 administration. **I.** Quantification of scar length as absolute mm. **J.** Quantification of average scar thickness as absolute mm. **K.** Quantification of left ventricle (LV) area as absolute mm^2. **L.** Quantification of LV average septum thickness as absolute mm. **M.** %FS at 3 dpi for control and IL4Rα^{MacKO} mice treated with rIL13, open circles represent mice that died before 28 dpi. Data shown as mean ±SD. Each data point represents one mouse. Darker data points represent male mice in A-E and G,H. *P<0.05, **P<0.01, ***P<0.001 and ****P<0.0001. Genotype effect by 2w ANOVA and Sidak's post-hoc comparison in B, D and I. Interaction effect between genotype and treatment by 2w ANOVA and Sidak's post-hoc comparison in C and G. Interaction effect between time and genotype by 2w RM ANOVA and Sidak's post-hoc comparison for rIL13 treated cohorts in F. Time effect by 2w RM ANOVA and Sidak's post-hoc comparison for PBS treated cohorts in F and both cohorts in H. Treatment effect by 2w ANOVA in J. Comparison by 2w ANOVA in K and L. Comparison by t-test in M.

Figure S5

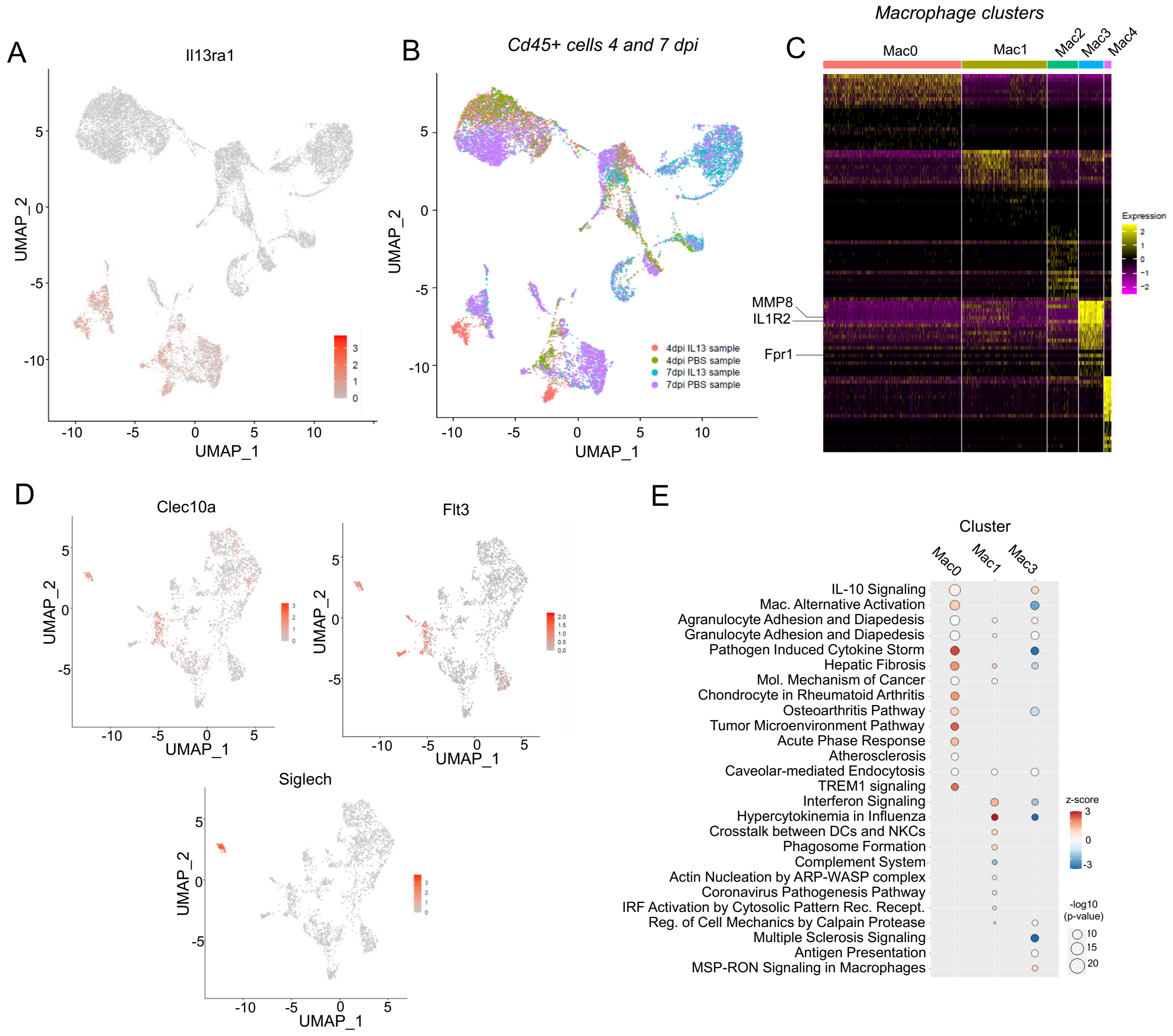

A. Feature plot of IL13Rα1 from all leukocyte clustering corresponding to Fig. 6A. Scalebar represents gene expression level **B.** UMAP plot of cell distribution by sample of all leukocytes corresponding to Fig. 6A. **C.** Heatmap showing most upregulated genes in each macrophage cluster (0 to 4) corresponding to clusters in Fig. 6D and Table S3. Color scalebar represents directionality of expression enrichment. **D.** Feature plots of Clec10a, Flt3, and Siglech from macrophage clustering corresponding to Fig. 6D. **E.** Ingenuity pathway analysis identified biologic processes that are uniquely activated in macrophage

clusters 0, 1 and 3 from figure 6D. Color scalebar represents directionality and circle size represents statistical significance.

Figure S6

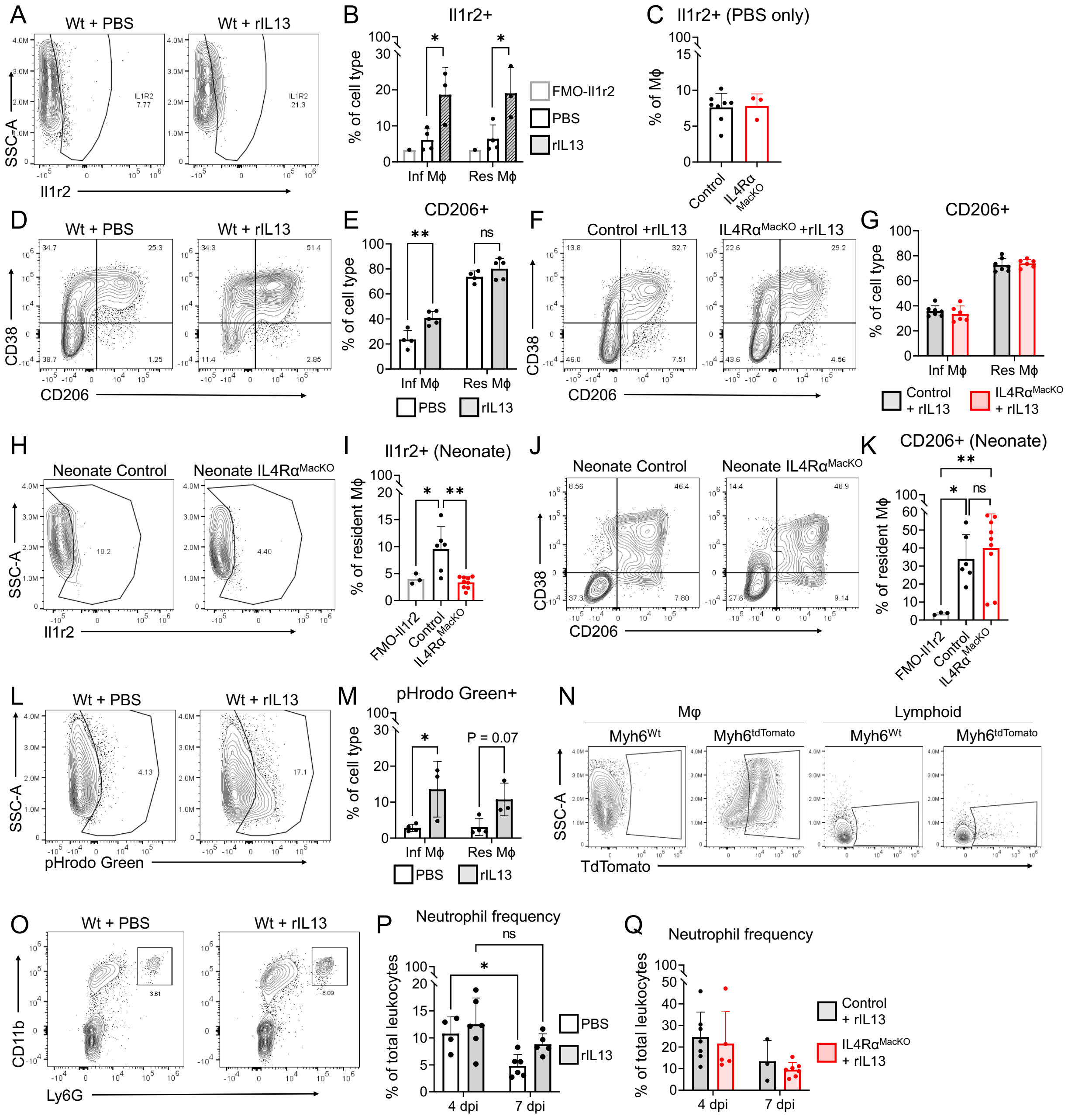

A. Representative contour plots showing Il1r2 expression in infiltrating macrophages of adult C57BL6/j (Wt) mice treated with PBS vs. rIL13 at 4 dpi after MI. **B.** Quantification of cardiac Il1r2+ macrophage frequency at 4 dpi after MI. **C.** Quantification of cardiac Il1r2+ macrophage frequency in PBS-treated adult control and IL4Rα^{MacKO} hearts at 4 dpi after

MI. **D.** Representative contour plots of CD38 and CD206 expression in infiltrating macrophages from Wt mice at 4 dpi after MI and rIL13 vs. PBS administration. **E.** Quantification of cardiac CD206+ macrophage frequency in adult Wt mice at 4 dpi after MI and PBS vs. rIL13 administration. **F.** Representative contour plots of CD38 and CD206 expression in cardiac infiltrating macrophages from adult control and L4Rα^{MacKO} mice at 4 dpi after MI and rIL13 administration. **G.** Quantification of cardiac CD206+ macrophage frequency in adult control or L4Rα^{MacKO} mice at 4 dpi after MI and rIL13 administration. **H.** Representative contour plots of Il1r2 expression in cardiac resident macrophages in neonatal control and L4Rα^{MacKO} mice at 4 dpi after P1 MI. **I.** Quantification of cardiac Il1r2+ macrophage frequency in neonatal control and L4Rα^{MacKO} mice at 4 dpi after P1 MI. **J.** Representative contour plots of CD38 and CD206 expression in cardiac resident macrophages in neonatal control and L4Rα^{MacKO} mice at 4 dpi after P1 MI. **K.** Quantification of cardiac CD206^{+} macrophage frequency in neonatal control and L4Rα^{MacKO} mice at 4 dpi after P1 MI. **L.** Representative contour plots showing pHrodo green E. Coli signal in infiltrating cardiac macrophages in Wt mice at 4 dpi after MI and PBS vs. rIL13 administration. **M.** Quantification of pHrodo green E. Coli+ macrophage frequency in adult Wt mice at 4 dpi after MI and PBS vs. rIL13 administration. **N.** Representative contour plots showing tdTomato signal in cardiac macrophages and lymphoid cells in Myh6^{tdTomato} and Myh6^{Wt} littermate mice. **O.** Representative contour plots showing cardiac neutrophils population in Wt mice at 7 dpi after MI and PBS vs. rIL13 administration. **P.** Quantification of cardiac neutrophils frequency in Wt mice at 4 and 7 dpi after MI and PBS vs. rIL13 administration, and **(Q)** control and L4Rα^{MacKO} mice after MI and rIL13 administration. Data shown as mean ±SD. Each datapoint represents one

mouse. *P<0.05 and **P<0.01. Treatment effect by 2w RM ANOVA and Sidak's post-hoc comparison in B and M. Comparison by t-test in C. Interaction effect of cell type and treatment by 2w RM ANOVA and Sidak's post-hoc comparison in E. Comparison by 2w RM ANOVA in G. Comparison by 1w ANOVA and Tukey's post-hoc test in I and K. Interaction effect between time and treatment by 2w ANOVA and Sidak's post-hoc comparison in P. Comparison by 2w ANOVA in Q.

1. Herbert, D.R., et al., *Alternative macrophage activation is essential for survival during schistosomiasis and downmodulates T helper 1 responses and immunopathology.* Immunity, 2004. **20**(5): p. 623-35.
2. Paddock, S.J., et al., *IL4Ralpha signaling promotes neonatal cardiac regeneration and cardiomyocyte cell cycle activity.* J Mol Cell Cardiol, 2021.
3. Porrello, E.R., et al., *Transient regenerative potential of the neonatal mouse heart.* Science, 2011. **331**(6020): p. 1078-80.
4. Patterson, M., et al., *Frequency of mononuclear diploid cardiomyocytes underlies natural variation in heart regeneration.* Nat Genet, 2017. **49**(9): p. 1346-1353.
5. Wodsedalek, D.J., et al., *IL-13 promotes in vivo neonatal cardiomyocyte cell cycle activity and heart regeneration.* Am J Physiol Heart Circ Physiol, 2019. **316**(1): p. H24-H34.
6. Nascimento, D.S., et al., *MIQuant--semi-automation of infarct size assessment in models of cardiac ischemic injury.* PLoS One, 2011. **6**(9): p. e25045.
7. Stuart, T., et al., *Comprehensive Integration of Single-Cell Data.* Cell, 2019. **177**(7): p. 1888-1902 e21.
8. Hafemeister, C. and R. Satija, *Normalization and variance stabilization of single-cell RNA-seq data using regularized negative binomial regression.* Genome Biol, 2019. **20**(1): p. 296.
9. Franzen, O., L.M. Gan, and J.L.M. Bjorkegren, *PanglaoDB: a web server for exploration of mouse and human single-cell RNA sequencing data.* Database (Oxford), 2019. **2019**.
